# Disruptor of telomeric silencing 1-like promotes ovarian cancer tumor growth by stimulating pro-tumorigenic metabolic pathways and blocking apoptosis

**DOI:** 10.1038/s41389-021-00339-6

**Published:** 2021-07-12

**Authors:** Suresh Chava, Suresh Bugide, Yvonne J. K. Edwards, Romi Gupta

**Affiliations:** Department of Biochemistry and Molecular Genetics, The University of Alabama at Birmingham, Birmingham, AL 35233 USA

**Keywords:** Cancer genetics, Cancer genomics

## Abstract

Ovarian cancer is the leading cause of gynecological malignancy-related deaths. Current therapies for ovarian cancer do not provide meaningful and sustainable clinical benefits, highlighting the need for new therapies. We show that the histone H3K79 methyltransferase disruptor of telomeric silencing 1-like (DOT1L) is overexpressed in ovarian cancer and that a higher level of DOT1L expression correlates with shorter progression-free and overall survival (OS). Pharmacological inhibition of DOT1L (EPZ-5676, EPZ004777, and SGC0946) or genetic inhibition of DOT1L attenuates the growth of ovarian cancer cells in cell culture and in a mouse xenograft model of ovarian cancer. Transcriptome-wide mRNA expression profiling shows that DOT1L inhibition results in the downregulation of genes involved in cellular biosynthesis pathways and the upregulation of proapoptotic genes. Consistent with the results of transcriptome analysis, the unbiased large-scale metabolomic analysis showed reduced levels of several metabolites of the amino acid and nucleotide biosynthesis pathways after DOT1L inhibition. DOT1L inhibition also resulted in the upregulation of the NKG2D ligand ULBP1 and subsequent increase in natural killer (NK) cell-mediated ovarian cancer eradication. Collectively, our results demonstrate that DOT1L promotes ovarian cancer tumor growth by regulating apoptotic and metabolic pathways as well as NK cell-mediated eradication of ovarian cancer and identifies DOT1L as a new pharmacological target for ovarian cancer therapy.

## Introduction

Ovarian cancer is the leading cause of gynecological cancer-related deaths [1,2,]. Despite decades of research to develop new treatment modalities, the 5-year survival rate of patients with advanced ovarian cancer (stage III and IV) is between 10 and 30% [3,4,]. There is an urgent need for new therapies against ovarian cancer because resistance to therapies and disease recurrence are common [5,6,], and current therapies for patients with advanced ovarian cancer are largely ineffective [7,8,]. Epigenetic regulators such as chromatin modifiers play key roles in tumor growth, progression, and therapy response [9–11], but there is limited information about their role in ovarian cancer.

The chromatin modifier disruptor of telomeric silencing 1-like (DOT1L) methylates lysine 79 of nucleosomal histone H3 (H3K79) [12] to create an important epigenetic modification that affects many biological processes including telomeric silencing, cell cycle regulation, transcriptional activation, and DNA repair [13–15]. Previous studies highlighted the role of DOT1L in the development and maintenance of mixed lineage leukemia (MLL)-rearranged leukemia [16]. Subsequent studies of the effect of DOT1L inhibitors in patients with MLL-rearranged leukemia had promising results [17]. In ovarian cancer, DOT1L has been shown to promote tumor growth by associating with estrogen receptor alpha to regulate cell cycle progression, epithelial–mesenchymal transition, drug metabolism, and cell-to-cell signaling [18,19,].

In this study, we show that DOT1L is overexpressed in ovarian cancer and that this overexpression is linked to advanced tumor stage as patients with higher DOT1L expression exhibit shorter progression-free and overall survival (OS) than those with lower DOT1L expression. We also demonstrate that both pharmacological and genetic inhibition of DOT1L attenuates ovarian cancer growth in cell culture and in a mouse xenograft model. Mechanistically, DOT1L inhibition in ovarian cancer cells results in the downregulation of genes involved in cellular biosynthetic pathways and the upregulation of proapoptotic genes. Further, unbiased large-scale metabolomic analysis confirmed that DOT1L inhibition reduced the levels of several metabolites involved in amino acid, glycolytic, and nucleotide biosynthesis pathways as a result of downregulation of several genes involved in cellular biosynthetic pathways, as outcomes of DOT1L inhibition. Pharmacological inhibition of DOT1L additionally upregulates the expression of natural killer group 2 D (NKG2D) ligands, in particular ULBP1, which correlates with increased natural killer (NK) cell-mediated eradication of ovarian cancer cells. Taken together, our results demonstrate that DOT1L promotes ovarian cancer tumor growth and represents a new therapeutic target for ovarian cancer treatment.

## Results

### DOT1L is overexpressed in ovarian cancer and its overexpression is associated with a poor prognosis

**Fig. 1:**
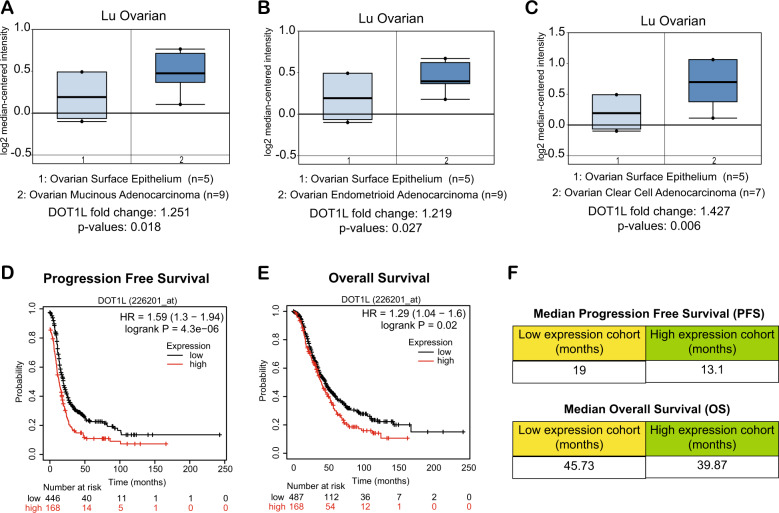
DOT1L is overexpressed in patient-derived samples of ovarian cancer. **A**–**C** The indicated ovarian cancer patient datasets were analyzed for *DOT1L* mRNA expression. Upregulation of *DOT1L* mRNA in ovarian cancer samples compared with normal ovarian surface epithelium cells is indicated. **D**, **E** The effects of DOT1L expression on progression-free survival and overall survival were analyzed using a Kaplan–Meyer plotter. **F** Median progression-free survival and overall survival of patients with low or high DOT1L expression.

To understand the role of DOT1L in ovarian cancer, we first asked if DOT1L expression is altered in ovarian cancer, and if so, whether its expression is associated with disease outcomes. Analysis of publicly available ovarian cancer datasets revealed that *DOT1L* mRNA was significantly higher in patient-derived samples of ovarian cancer than in corresponding normal tissues [20] (Fig. 1A–C). Furthermore, patients with ovarian tumors with high DOT1L expression had shorter progression-free survival (PFS) and (OS) than patients with ovarian tumors with low DOT1L expression (Fig. 1D, E). The median PFS of patients with low DOT1L expression was 19 months, whereas that of patients with high DOT1L expression was only 13.1 months. Similarly, the median OS of patients with low DOT1L expression was 45.73 months, whereas that of patients with high DOT1L expression was 39.87 months (Fig. 1F). Collectively, those results indicated that DOT1L is overexpressed in ovarian cancer and that its overexpression is associated with poor prognosis.

### DOT1L inhibition blocks the growth of ovarian cancer cells in vitro and in a xenograft model of ovarian cancer

**Fig. 2:**
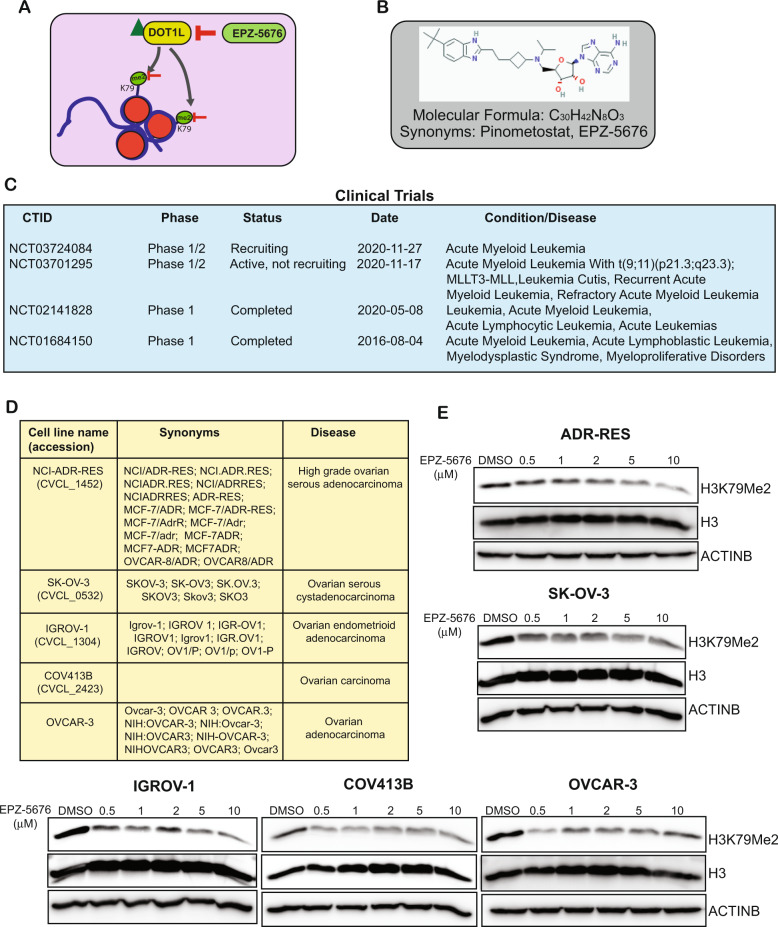
The DOT1L inhibitor EPZ-5676 inhibits H3K79me2 mark in ovarian cancer cell lines. **A** Schematics showing how DOT1L regulates the H3K79 dimethyl mark and how the pharmacological inhibition of DOT1L via EPZ-5676 leads to suppression of the mark. **B** EPZ-5676 inhibitor structure and formula. **C** EPZ-5676 inhibitor studies in clinical trials as reported on ClinicalTrials.gov. **D** The common names, synonyms, and disease associations of the ovarian cancer cell lines used in this study. **E** The indicated ovarian cancer cell lines were treated with various concentrations of the DOT1L inhibitor EPZ-5676 for 48 h. H3K79 dimethyl marks were then measured. Histone H3 and ACTINB proteins were measured as loading controls.

DOT1L methylates lysine 79 of nucleosomal histone H3 (H3K79) and EPZ-5676, (also known as Pinometostat) is an *S*-adenosyl methionine competitive inhibitor of DOT1L. EPZ-5676 has an inhibitory constant (Ki) of 80 pM in a cell-free assay and has demonstrated >37,000-fold selectivity for DOT1L relative to all the other protein methyltransferases tested [21] (Fig. 2A, B). In cell culture, EPZ-5676 inhibited H3K79 methylation and MLL-fusion gene expression and selectively inhibited the growth of acute leukemia cell lines bearing MLL translocations [21]. Similarly, in a rat xenograft model of MLL-rearranged leukemia, EPZ-5676 treatment resulted in complete and sustained tumor regression even after the treatment was stopped [21]. EPZ-5676 also showed synergistic antiproliferative activity in combination with standard-of-care drugs and hypomethylating agents in MLL-rearranged leukemia cells [17]. Because DOT1L plays an important role in the development and maintenance of MLL-rearranged leukemia, EPZ-5676 has been used in several clinical trials for the treatment of the same (Fig. 2C).

**Fig. 3:**
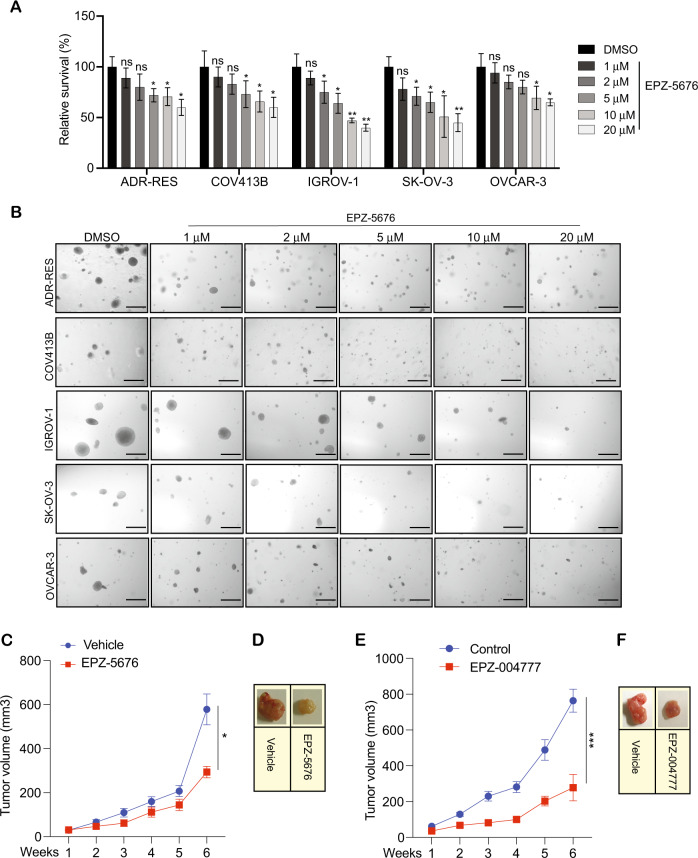
Pharmacological inhibition of DOT1L inhibits the growth of ovarian cancer cells. **A** The indicated ovarian cancer cell lines were treated with the different concentrations of DOT1L inhibitor EPZ-5676 for 3 days and analyzed for cell survival in MTT assays. Relative cell survival is plotted with respect to control treated cells. **B** The indicated ovarian cancer cell lines were treated with various concentrations of EPZ-5676 and analyzed for the ability to grow in an anchorage-independent manner in soft agar assays. Representative images of soft agar assays are shown. Scale bar, 500 μm**. C**, **D** IGROV-1 cells were injected subcutaneously into the flanks of NSG mice (*n* = 7). The mice were treated with EPZ-5676 (50 mg/kg) intraperitoneally every day and analyzed for tumor growth. Tumor sizes were measured each week and the average tumor volume is plotted (**C**). Representative images of the tumors from the mice after 6 weeks of EPZ-5676 treatment are shown (**D**). **E**, **F** IGROV-1 cells were injected subcutaneously into the flanks of NSG mice (*n* = 5). The mice were treated with EPZ004777 (50 mg/kg) intraperitoneally every day and analyzed for tumor growth. Tumor sizes were measured each week and the average tumor volume is plotted (**E**). Representative images of the tumors from the mice after 6 weeks of EPZ004777 treatment are shown (**F**). Data were shown as the mean ± SEM, *p < 0.05, **p < 0.01, ***p < 0.001, ns not significant, calculated using the Student’s *t*-test.

In our study, we used the drug EPZ-5676 to determine if DOT1L is necessary for the growth of ovarian cancer cells and whether its inhibition can suppress their growth. To this end, we treated COV-413B, ADR-RES, OVCAR-3, IGROV-1, and SK-OV-3 ovarian cancer cells with several concentrations of EPZ-5676 for 48 h and then assayed the cells for the H3K79 dimethyl (H3K79me2) marks (Fig. 2D). The results showed that multiple ovarian cancer cells that were treated with EPZ-5676 had reduced levels of H3K79me2 marks compared with DMSO-treated cells (Fig. 2E). We next tested the effect of EPZ-5676 on the short-term growth of ovarian cancer cells using MTT assays. For these experiments, we treated the ovarian cancer cell lines (ADR-RES, SK-OV-3, IGROV-1, COV-413B, and OVCAR-3) with EPZ-5676 and measured cell viability by MTT assay after 3 days of treatment. The results showed that EPZ-5676 significantly inhibited the survival of ovarian cancer cells (Fig. 3A). Next, we tested the effect of EPZ-5676 on the long-term growth of ovarian cancer cells using soft agar assays, which are commonly used as a surrogate assay to measure the tumor-forming potential of cancer cells [22,23,]. To this end, we treated the ovarian cancer cell lines (ADR-RES, SK-OV-3, IGROV-1, COV-413B, and OVCAR-3) with EPZ-5676 and measured their ability to form colonies in soft agar. The results showed that EPZ-5676 significantly inhibited the growth of ovarian cancer cells in a concentration-dependent manner (Fig. 3B and Supplementary Fig. 1).

In order to ascertain the specificity of the growth inhibitory effects of EPZ-5676 in the ovarian cancer cells, we also examined the effects of two other DOT1L-specific inhibitors, EPZ004777 and SGC0946. Both EPZ004777 and SGC0946 are potent and selective DOT1L inhibitors with an IC50 of 0.4 and 0.3 nM, respectively in a cell-free assay. Both of these inhibitors selectively inhibit cellular H3K79 methylation and suppresses the cancer cells proliferation [24–27]. We found that similar to EPZ-5676, both EPZ004777 and SGC0946 effectively inhibited the H3K79 dimethyl (H3K79me2) mark and suppressed short-term growth of the ovarian cancer cells in the MTT assay (Supplementary Fig. 2). They also effectively inhibit the ability of multiple ovarian cancer cell lines (SK-OV-3 and IGROV-1) to form colonies in soft agar (Supplementary Fig. 3).

We expressed *DOT1L* shRNA in ovarian cancer cells to confirm our results with EPZ-5676, EPZ004777, and SGC0946 treatment. These experiments revealed that, similar to pharmacological inhibition, genetic knockdown of DOT1L leads to a similar phenotype (Supplementary Fig. 4A–D).

In order to understand, whether the EPZ-5676-mediated growth inhibition of ovarian cancer cells growth is dependent on the expression levels of DOT1L and H3K79me2 chromatin mark, we measured their levels in a variety of ovarian cancer cells. Our results showed that the cell line, IGROV-1, which expressed the highest levels of DOT1L and H3K79me2 chromatin mark, was the most sensitive to EPZ-5676 mediated growth inhibition (Supplementary Fig. 5). Thus, the growth inhibitory effect of the DOT1L inhibitor is dependent on the expression levels of DOT1L and H3K79me2 chromatin mark. Together, our results showed that DOT1L is required for the growth of ovarian cancer cells and that its inhibition leads to the suppression of growth and tumor-forming characteristics in vitro.

After confirming the growth inhibitory effect of EPZ-5676 on ovarian cancer cells in vitro, we next examined if EPZ-5676 could inhibit the growth of ovarian cancer cells in a mouse xenograft model. To do so, we administered IGROV-1 ovarian cancer cells subcutaneously into the flanks of immunocompromised mice (NSG mice) and measured the effect of EPZ-5676 treatment on subcutaneous tumor growth. The results showed that EPZ-5676 significantly blocked subcutaneous ovarian cancer tumor growth in vivo in the xenograft model compared to vehicle-treated mice (Fig. 3C, D). Similar effects on ovarian cancer tumor growth in vivo in the xenograft model were observed with the administration of other DOT1L inhibitors, EPZ004777 (Fig. 3E, F) and *DOT1L* shRNA (Supplementary Fig. 6). Collectively, these results demonstrate that DOT1L inhibition effectively attenuates the growth of ovarian cancer cells, suggesting that DOT1L pharmacological inhibition can be employed for the treatment of ovarian cancer.

### DOT1L inhibition leads to activation of cell death pathways and suppression of cellular biosynthesis pathways in ovarian cancer cells

**Fig. 4:**
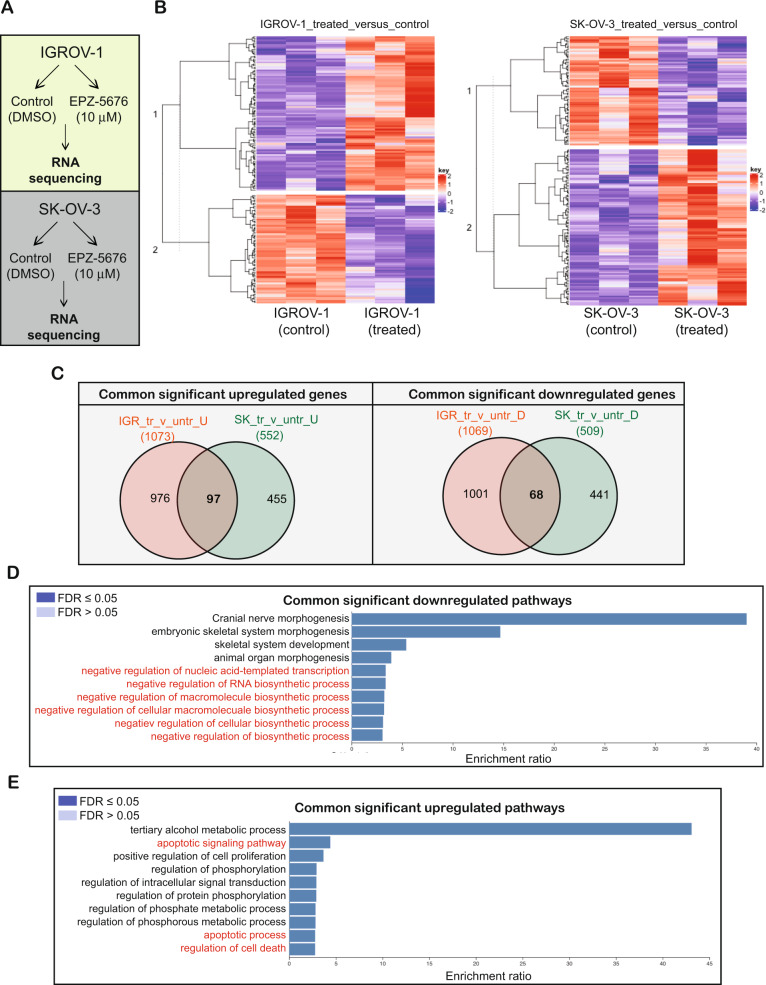
Pharmacological inhibition of DOT1L leads to the alteration of multiple genes that affect the growth of ovarian cancer cells. **A** Schematics of the RNA sequencing analysis performed with IGROV-1 and SK-OV-3 cells. **B** Heatmaps show the overall alterations of gene expression in IGROV-1 and SK-OV-3 cells treated with 10 μM EPZ-5676 for 48 h in comparison with control cells. **C** Venn diagrams show the genes that were significantly upregulated (*n* = 97) or downregulated (*n* = 68) in both cell lines (IGROV-1 and SK-OV-3) after treatment with 10 μM EPZ-5676 for 48 h. **D** A bar diagram shows the pathways that were downregulated in both ovarian cancer cell lines after treatment with 10 μM EPZ-5676 for 48 h. **E** A bar diagram shows the pathways that were upregulated in both ovarian cancer cell lines after treatment with 10 μM EPZ-5676 for 48 h.

We next performed experiments to determine the mechanism by which DOT1L inhibition attenuates ovarian cancer cell growth. Because DOT1L is a chromatin modifier, we hypothesized that it promotes ovarian cancer growth by regulating gene expression. To test that, we treated two ovarian cancer cell lines, IGROV-1 and SK-OV-3, with EPZ-5676 and performed transcriptome-wide RNA sequencing (RNA-seq) analysis (Fig. 4A). We identified several genes that were differentially expressed after EPZ-5676 treatment (Fig. 4B, and Supplementary Tables 1, 2). Overall, 97 genes were upregulated in both cell lines after EPZ-5676 treatment, whereas 68 genes were downregulated in both cell lines (Fig. 4C, Supplementary Fig. 7, and Supplementary Tables 3, 4).

A pathway analysis revealed that several of the genes that were upregulated after EPZ-5676 treatment were involved in cell death signaling/apoptotic pathways, including AKR1C3, ATF3, BMP2, CCND2, CHAC1, DAPK2, DDIT3, DDIT4, GADD45A, GSTP1, IL1A, INHBE, IRF1, IRS2, JUN, NQO1, OSGIN1, PEA15, PIM3, PPP1R15A, SERPINE1, SPHK1, SQSTM1, STK40, TNFAIP3, TNFRSF21, TRIB3, UNC5B, and VEGFA (Fig. 4D, Supplementary Fig. 8, and Supplementary Table 5). Additionally, several of the genes that were downregulated in both cell lines after EPZ-5676 treatment; including ARID5B, EZH2, HMGA2, HOXB3, HOXB4, IGF2BP2, KCTD1, LGR4, MECOM, MEIS2, MIER1, NR2F2, RBPJ, TFAP2A, ZFP36L2, ZMYND8, ZNF217, HOXB2, HOXB5, PDGFC, and LBH; participate in cellular biosynthesis pathways such as macromolecule biosynthesis, RNA biosynthesis, organ development, skeletal system morphogenesis, and stem cell division (Fig. 4E, Supplementary Fig. 9, and Supplementary Table 6). These results suggest that DOT1L facilitates tumor growth and metastasis by upregulating tumor-promoting biosynthetic pathways and inhibiting proapoptotic pathways in ovarian cancer cells.

### The DOT1L inhibitor EPZ-5676 promotes cell death in ovarian cancer cells

**Fig. 5:**
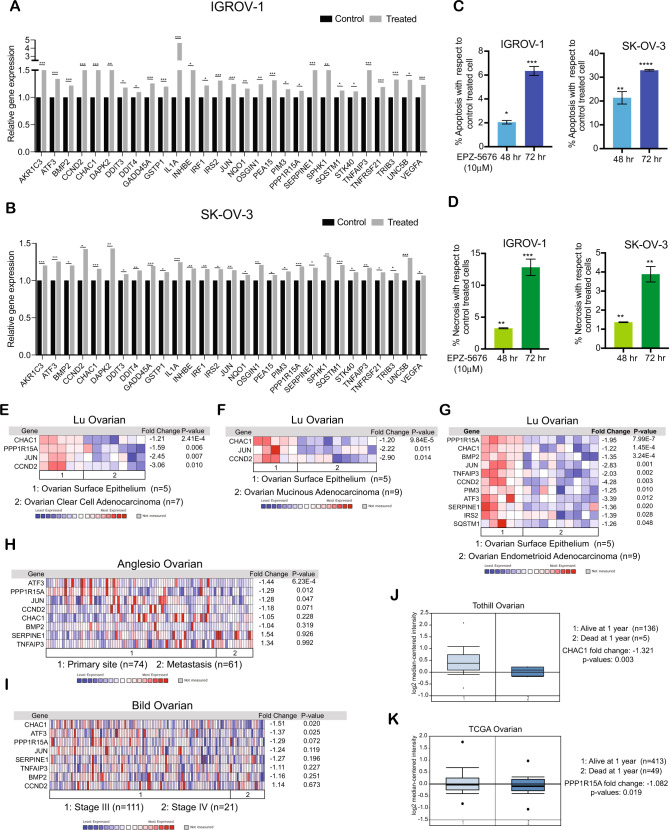
Pharmacological inhibition of DOT1L leads to the upregulation of multiple genes involved in apoptotic and cell death pathways. **A**, **B** Expression of candidate genes involved in apoptotic and cell death pathways in IGROV-1 and SK-OV-3 cells treated with 10 μM EPZ-5676 for 48 h relative to that in control cells. **C** A bar diagram shows the apoptosis in IGROV-1 and SK-OV-3 cells treated with 10 μM EPZ-5676 for 48 and 72 h as a percentage in comparison with the control treated cells. **D** A bar diagram shows the necrosis in IGROV-1 and SK-OV-3 cells treated with 10 μM EPZ-5676 for 48 and 72 h as a percentage in comparison with the control treated cells. **E**–**G** The Lu ovarian cancer patient datasets were analyzed for apoptotic gene expression. The fold expression of candidate genes with *p* values <0.05 are shown. **H** The Anglesio ovarian cancer patient datasets were analyzed for apoptotic gene expression at primary tumor sites and metastatic sites. The fold expression of candidate genes with *p* values <0.05 are shown. **I** The Bild ovarian cancer patient datasets were analyzed for apoptotic gene expression in samples of stage III and stage IV ovarian cancer. The fold expression of candidate genes with p values <0.05 are shown. **J** CHAC1 expression was analyzed in the Tothill ovarian cancer patient datasets. **K** PPP1R15A expression was analyzed in The Cancer Genome Atlas ovarian cancer patient datasets. Data were shown as the mean ± SEM, *p < 0.05, **p < 0.01, ***p < 0.001, ****p < 0.0001, ns not significant, calculated using the Student’s *t*-test.

A large number of the genes that were upregulated as a result of EPZ-5676 treatment in ovarian cancer cells were proapoptotic (Fig. 5A, B and Supplementary Fig. 10A, B). Upregulation of many of the proapoptotic genes identified in EPZ-5676- treated ovarian cancer cells was confirmed in cells expressing *DOT1L* shRNA. We, therefore, investigated if the treatment of ovarian cancer cells with EPZ-5676 induces apoptosis by measuring cell death (apoptosis and necrosis) in IGROV-1 and SK-OV-3 cells after treatment with EPZ-5676. The results showed that in both cell lines, EPZ-5676 treatment promoted cell death (Fig. 5C, D). Additionally, similar results were obtained in *DOT1L* shRNA expressing ovarian cancer cells (Supplementary Fig. 11 A–C).

To determine if the regulation of proapoptotic genes by DOT1L is clinically relevant, we checked whether the proapoptotic genes that were upregulated as a result of EPZ-5676 treatment in the ovarian cancer cell lines were repressed in ovarian tumor samples in which DOT1L was overexpressed. We found that the expression of many of the proapoptotic genes was significantly downregulated in the ovarian tumor samples relative to that in matched samples of the normal ovarian surface epithelium [20] (Fig. 5E, F, G and Supplementary Fig. 11D). We also checked whether the downregulation of any of the proapoptotic genes was associated with metastasis or advanced tumor stage in the patient-derived samples. We found that ATF3, PPP1R15A, and JUN downregulation was associated with metastasis [28] (Fig. 5H), and CHAC1 and ATF3 downregulation was associated with stage IV disease [29] (Fig. 5I). In addition, CHAC1 and PPP1R15A downregulation was associated with shorter OS in ovarian cancer patients [30] (Fig. 5J, K). These results demonstrate that DOT1L regulates the expression of clinically significant proapoptotic genes and pathways that determine the severity of ovarian cancer.

### An unbiased large-scale metabolomic analysis revealed alterations in the amino acid and nucleotide metabolic pathway in EPZ-5676 treated ovarian cancer cells

**Fig. 6:**
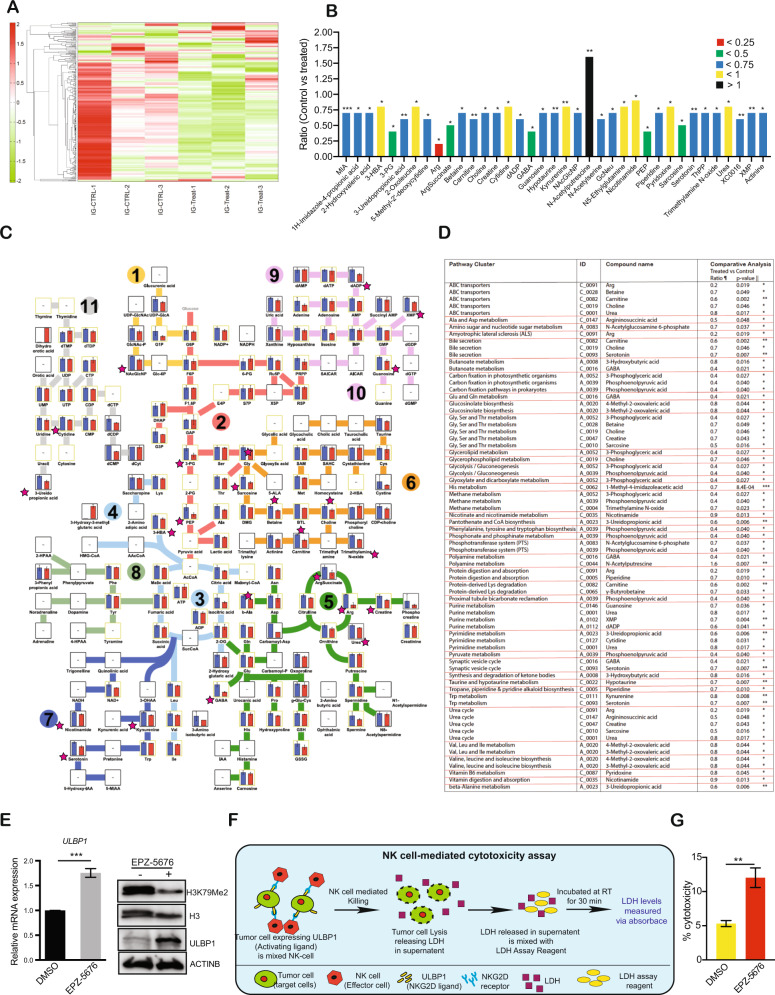
Pharmacological inhibition of DOT1L inhibits ovarian cancer growth in vivo. **A** Heatmaps show the overall metabolite alterations in IGROV-1 cells treated with 10 μM EPZ-5676 for 48 h in comparison with control cells. **B** Expression level of metabolite that were significantly altered in IGROV-1 cells treated with 10 μM EPZ-5676 for 48 h relative to that in control cells. **C** Diagram showing the metabolic pathways that were measured in global large-scale metabolomics analysis along with the significantly altered metabolites, which are indicated with a pink star. **D** Metabolic pathways that were associated with significantly altered metabolites in IGROV-1 cells treated with 10 μM EPZ-5676 for 48 h. **E** ULBP1 mRNA and protein expression in IGROV-1 cells treated with 10 μM EPZ-5676 for 48 h compared with that in control cells. **F** Schematics of NK cell-mediated cytotoxic assay. **G** Bar diagram showing the percentage of NK cell cytotoxicity in IGROV-1 cells treated with 10 μM EPZ-5676 for 48 h compared with the cytotoxicity in control treated cells. Data were shown as the mean ± SEM, *p < 0.05, **p < 0.01, ***p < 0.001, ns not significant, calculated using the Student’s *t*-test.

Because we observed, several of the genes that were downregulated after EPZ-5676 treatment in ovarian cancer cells are associated with cellular biosynthesis pathways such as macromolecule biosynthesis, RNA biosynthesis, organ development, skeletal system morphogenesis, and stem cell division we performed a large-scale unbiased metabolomic analysis with the goal of understanding the specific mechanism of DOT1L action. To do so, IGROV-1 cells were treated with EPZ-5676 and were analyzed using capillary electrophoresis time-of-flight mass spectrometry in two modes, for cationic and anionic metabolites. We detected 278 different metabolites from different metabolic pathways, out of which 37 of them were significantly altered in control versus treated samples (Fig. 6A, B and Supplementary Table 7). We found that these significantly altered metabolites participate in multiple pathways such as amino acid, glycolytic, and nucleotide biosynthetic pathways (Fig. 6C, D). In particular, the metabolites for which the ratio of control versus treated samples was less than 0.5 included Arg, ArgSuccinate (Argininosuccinic acid), GABA (gamma-Aminobutyric acid), PEP (Phosphoenolpyruvic acid), and Sarcosine. Arg, ArgSuccinate, and GABA have been shown to be part of glutamate metabolism [31] and the urea cycle, which has been shown to be important for cancer growth [32]. PEP is also needed for various anabolic processes such as carbohydrate metabolism/glycolysis and the gluconeogenesis/TCA cycle for energy storage and energy conversion to beta oxidation and BCAA metabolism. Sarcosine is known to be involved in pathways such as glycolysis, gluconeogenesis/choline metabolism, and the methionine salvage pathway. Thus, PEP and sarcosine metabolites manage a wide range of regulatory functions thus contributing to tumor progression by affecting various aspects of tumor biology [33–36]. Collectively, our metabolomic analysis confirmed that DOT1L inhibition negatively regulates several genes involved in cellular biosynthesis, thereby decreasing the level of several metabolites in ovarian cancer cells. Thus, DOT1L plays important roles in the regulation of ovarian cancer tumor growth and progression.

### The DOT1L inhibitor EPZ-5676 upregulates the activation of NKG2D ligands

Because EPZ-5676 was a potent inhibitor of tumor growth and metastasis in the mouse xenograft model of ovarian cancer, we reanalyzed the RNA sequencing data from EPZ-5676–treated IGROV-1 and SK-OV-3 cells. We found that in addition to the genes that regulate cellular biosynthetic and proapoptotic signaling pathways, the expression of several NKG2D ligands; including ULBP1, ULBP2, ULBP3, MICA, and MICB were also altered as a result of EPZ-5676 treatment. We revalidated our RNA sequencing data by performing quantitative RT-PCR and found that different sets of NKG2D ligands were upregulated in each cell line after EPZ-5676 treatment (Supplementary Fig. 12). ULBP1 was the only NKG2D ligand that was significantly upregulated at the mRNA level in multiple ovarian cancer cell lines (Fig. 6E and Supplementary Figs. 12, 13A). Next, we measured the ULBP1 protein expression in all of the ovarian cancer cell lines. The results showed that ULBP1 protein expression was upregulated after EPZ-5676 treatment only in IGROV-1 cells but not in the other cell lines (Fig. 6E). Analysis of patient-derived samples of ovarian cancer showed that the intensity and quantity of ULBP1 expression were lower in samples with high DOT1L expression than in samples with low DOT1L expression (Supplementary Fig. 13 B, C).

We next asked whether the level of ULBP1 expression affected NK cell-mediated killing of ovarian cancer cells. After treating IGROV-1 cells with EPZ-5676, we assayed NK cell-mediated killing of the cells as described previously [37]. We found that EPZ-5676 treatment resulted in increased NK cell-mediated killing of the IGROV-1 cells (Fig. 6F, G). Together, our results indicated that in a subset of ovarian cancer cell lines, DOT1L represses the expression of the activating NK cell ligand ULBP1, resulting in suppression of NK cell antitumor function.

## Discussion

**Fig. 7:**
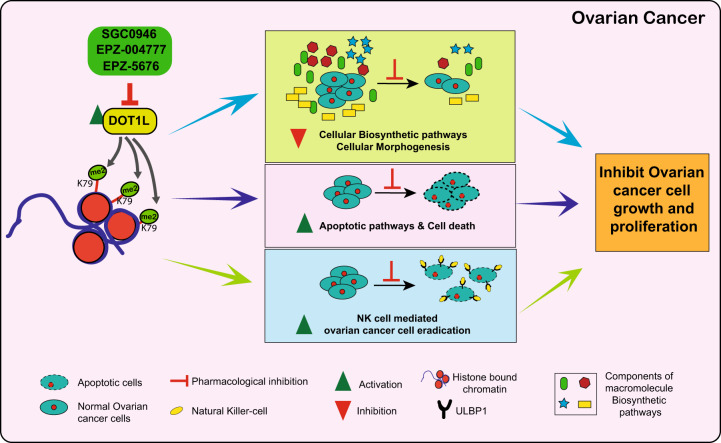
A model of DOT1L function in ovarian cancer. DOT1L promotes ovarian cancer growth and metastasis by upregulating cellular biosynthesis and morphogenesis pathways, downregulating apoptotic and cell death signaling pathways, and inhibiting NK cell-mediated ovarian cancer cell eradication.

Epigenetic regulators such as chromatin modifiers play important roles in the development and progression of cancer. DOT1L is a chromatin modifier that functions by acting as an H3K79me2 transferase, which we show is overexpressed in ovarian cancer, and its overexpression is predictive of poor prognosis. DOT1L was also necessary for ovarian cancer tumor growth in both cell culture and in mice. Mechanistically, we show that DOT1L activates the expression of genes promoting cellular biosynthesis and suppresses the expression of genes promoting cell death. Consistent with the results from transcriptome analysis, the unbiased large-scale metabolomic analysis showed DOT1L inhibition reduced levels of several metabolites involved in the amino acid and nucleotide biosynthesis pathways after DOT1L inhibition. Finally, we find that DOT1L can suppress NK cell-mediated anti-ovarian cancer immunity, in part by repressing NK cell-activating ligands, such as ULBP1. Our major findings are summarized in the model shown in Fig. 7 and discussed below.

DOT1L was previously reported to be overexpressed in several cancer types [38–40]. In prostate cancer, DOT1L overexpression correlates with disease progression [40] and it was suggested that it can be used as a biomarker for early detection [40]. Similarly, DOT1L is overexpressed and plays important role in gastric cancer [38], triple-negative breast cancer [41], and clear-cell renal carcinoma [42]. We showed that the DOT1L inhibitors (EPZ-5676, EPZ004777, and SGC0946) reduces the H3K79me2 mark and inhibit the growth of multiple ovarian cancer cell lines. Similar results were obtained in an ovarian cancer cell line expressing DOT1L shRNA. Our RNA sequencing results showed that the treatment of ovarian cancer cells with EPZ-5676 inhibits the expression of several genes involved in cellular biosynthesis pathways. An unbiased large-scale metabolomic analysis in EPZ-5676-treated ovarian cancer cells showed similar results, confirming the changes in metabolites due to the downregulation of several metabolic genes. For example, some of the genes that were downregulated included *N*-acetylglutamate synthase (NAGS) and glutamate decarboxylase 1 (GAD) shown to be involved in regulating metabolite Arg; glucose-6-phosphate isomerase (GPI), fructose-bisphosphate aldolase A (ALDOA), glyceraldehyde-3-phosphate dehydrogenase (GAPDH), phosphoglycerate kinase 1 (PGK1), phosphoglycerate kinase 1 (PGM1) shown to be involved in regulating metabolite 3-phosphoglyceric acid and phosphoenolpyruvic acid; serine hydroxymethyltransferase 1 (SHMT1) and glycine *N*-methyltransferase (GNMT) shown to be involved in regulating metabolite sarcosine etc. The upregulation of cellular biosynthesis pathways [43–46] play important roles in tumor development and progression and are considered to be important cancer hallmarks. Numerous studies have demonstrated that cancer cells support their high growth rates by activating oncogenic signaling pathways that lead to increased nutrient uptake and metabolism [43,47,]. Thus, amino acid, glycolytic, and nucleotide metabolic pathways have been successfully targeted in cancer treatments [48–50].

Our RNA sequencing results also showed that the treatment of ovarian cancer cells with EPZ-5676 activates the expression of several proapoptotic genes indicating that DOT1L downregulates the expression of several of the cell death-inducing genes, evading apoptosis and cell death to promote cancer growth and progression [51]. Similar results were obtained in an ovarian cancer cell line expressing DOT1L shRNA. We also found that some of the proapoptotic genes whose expression was increased in ovarian cancer cells after EPZ-5676 treatment were associated with disease progression and metastasis in patients with ovarian cancer. For example, the downregulation of CHAC1 and PPP1R15A were associated with early mortality in ovarian cancer patients [20]. Thus, inhibition of cell death-promoting pathways [52–56] plays important roles in tumor development and progression, making this an important hallmark of cancer. In summary, our results suggest that DOT1L-regulated pro-apoptosis genes play an important role in ovarian cancer and is associated with poor prognosis.

Cancer cells harbor genetic and epigenetic alterations that generate neoantigens that can be recognized by the host immune system [57]. Tumors exploit several distinct pathways to evade immune detection, including immune checkpoints that terminate the immune response after antigen activation. Intensive efforts are underway to develop treatments that inhibit immune checkpoint pathways with agents such as anti-CTLA-4 antibodies (ipilimumab) or anti-PD1/PD-L1 antibodies (pembrolizumab or nivolumab) [58]. Despite encouraging results in certain cancer types such as Hodgkin’s disease and melanoma [59–61], success has been limited in other cancer types, including ovarian cancer [3,62,]. NK cells are large granular lymphocytes of the innate immune system that protect humans against infectious agents and are the first line of immunological defense against tumor initiation. NK cells can spontaneously kill cancer cells and do not require pre-stimulation to exert their effector functions [63]. Following cytokine activation, NK cells can infiltrate most tissues that contain pathogens or tumor cells [64,65,]. It is not clear, however, how NK cell-mediated eradication of ovarian cancer cells is regulated. Studies have shown that genome-wide DNA methylation affects NK cell activation [66]. We found that pharmacological inhibition of DOT1L upregulates the expression of the NKG2D ligand ULBP1 in a subset of ovarian cancer cells, leading to NK cell-mediated killing of the ovarian cancer cells. These results suggest that DOT1L plays an important role in regulating the expression of NKG2D ligands and NK cell-mediated killing of ovarian cancer cells. EPZ-5676 is a highly selective and potent DOT1L inhibitor with proven efficacy in MLL-rearranged leukemia and is under clinical investigation. Our results suggest that DOT1L might be a pharmacologically tractable drug target for ovarian cancer therapy. It will also be useful in combination with other immunotherapeutic agents to further enhance their effectiveness in treating ovarian cancer.

## Experimental Methodology

### Cell culture and inhibitors

Ovarian cancer cell lines (COV-413B, IGROV-1, and SK-OV-3) were purchased from Sigma-Aldrich (St. Louis, MO, USA) OVCAR-3 was purchased from ATCC and ADR-RES was purchased from EZBiosystem and grown as recommended in RPMI-1640 or Dulbecco’s Modified Eagle Medium containing 10% FBS at 37 °C in 5% CO_2_. The DOT1L inhibitor Pinometostat (EPZ-5676), EPZ004777 and SGC0946 were purchased from Medchemexpress (MCE). Additional information is provided in Supplementary Table 8.

### DOT1 expression analysis in ovarian cancer gene expression datasets

Datasets with gene expression data from ovarian cancer tissues and normal ovarian surface epithelium tissues were identified by searching the Oncomine cancer profiling database. The Lu ovarian cancer dataset includes ovarian adenocarcinoma samples (*n* = 45) and normal ovarian surface epithelium samples (*n* = 5) analyzed on Affymetrix U95A-E microarrays [20]. The Tothill ovarian cancer dataset [20] includes samples from patients that were alive (*n* = 136) or not alive (*n* = 5) 1 year after diagnosis analyzed on Human Genome U133 Plus 2.0 Arrays [30]. The Anglesio ovarian dataset includes primary tumor samples (*n* = 74) and metastasis samples (*n* = 16) analyzed on Human Genome U133 Plus 2.0 Arrays [28]. The Bild ovarian cancer dataset includes Stage III (*n* = 111) and Stage IV (*n* = 21) tumor samples analyzed on Human Genome U133A Arrays [29]. The Cancer Genome Atlas ovarian cancer dataset includes samples from patients that were alive (*n* = 413) or not alive (*n* = 49) 1 year after diagnosis analyzed on Human Genome U133A Arrays.

### Isolation of mRNA and RT-qPCR analysis

Total RNA was extracted using TRIzol^®^ reagent (Invitrogen, Carlsbad, CA, USA) and purified with RNeasy mini columns (Qiagen, Hilden, Germany). The cDNA was generated using the M-MuLV first-strand cDNA synthesis kit (New England Biolabs, Ipswich, MA, USA) according to the manufacturer’s instructions. Quantitative reverse transcription polymerase chain reaction (RT-qPCR) was performed using the Power SYBR^®^ Green kit (Applied Biosystems, Foster City, CA, USA) according to the manufacturer’s instructions. Actin was used as an internal control. The primer sequences used in the study are provided in Supplementary Table 8.

### Sample preparation for RNA sequencing

IGROV-1 and SK-OV-3 cells treated with vehicle or 10 µM EPZ-5676 for 48 h were used to prepare total RNA, which was then used for gene expression analysis on an Illumina HiSeq 2500 system. Total RNA was extracted using TRIzol^®^ reagent (Invitrogen) according to the manufacturer’s instructions and then purified on RNAeasy mini columns (Qiagen) according to the manufacturer’s instructions. mRNA was purified from ~500 ng total RNA using oligo-dT beads and then sheared by incubation at 94 °C. Following first-strand synthesis with random primers, second-strand synthesis was performed with dUTP to generate strand-specific sequencing libraries. The cDNA libraries were then end-repaired and A-tailed. Adapters were ligated, and second-strand digestion was performed using Uracil-DNA-Glycosylase. Indexed libraries that met appropriate cut-offs for both were quantified by qRT-PCR using a commercially available kit (KAPA Biosystems, Wilmington, MA, USA). The insert size distribution was determined using LabChip GX or an Agilent Bioanalyzer. Samples with a yield ≥0.5 ng/μl were used for sequencing on the Illumina HiSeq 2500 system. Images generated by the sequencers were converted into nucleotide sequences by the base-calling pipeline RTA 1.18.64.0 and stored in FASTQ format.

### RNA sequencing data analysis

RNA sequencing was carried out for 12 samples comprising two cell lines (IGROV-1 and SK-OV-3) each with two treatment groups (control and treated) and three biological replicates per group. Single-end 75 bp reads were generated utilizing the Illumina NextSeq500 sequencing instrument. Pre-alignment quality assessments of the raw fastq sequences were carried out using FastQC (version 0.11.7) [67]. The number of reads for the 12 samples ranged from 31 to 48 M. The raw fastq sequences were aligned to the human hg38 reference genome (GenBank assembly accession: GCA_000001405.28) using STAR (version 2.7.1a) [68] with default parameters. Post-alignment quality assessments were carried out using RSeQC (version 2.6.3) [69]. Samtools (version 0.0.19) [70] and IGV (version 2.6.2) [71] were used to index and view the alignments, respectively. Gene expression was quantified as gene-level counts using the htseq-count function (version 0.12.3) [72] and the UCSC gene annotations for the human genome. The htseq-count default parameters were used, except for the strand parameter, which was set to “reverse” to account for the strandedness of the library. Genes for which there were less than three samples with normalized counts greater than or equal to four were filtered out. Differentially expressed genes were identified using DESeq2 (version 1.28) with default parameters [73]. Genes with a *p* value less than 0.05 were considered differentially expressed. InteractiVenn was used to generate Venn diagrams [74]. The normalized gene expression data were used for downstream analyses. The complex heatmap package version 1.12.0 [75] was used to generate heatmaps. To determine the cellular functions that were altered in both cell lines under the treatment conditions, overrepresentation enrichment analysis was performed using the WEB-based GEne SeT AnaLysis Toolkit (Webgestalt) [76] with the genome as the reference set and the Gene Ontology Biological Process database as the functional database. The hypergeometric test was used to test for the overrepresentation of functions among the differentially expressed genes common to both cell lines. The Benjamini and Hochberg method was used to calculate adjusted *p* values (*q*) with the significance cutoff filter set to *q* < 0.05.

### Immunoblot analysis

Whole-cell protein extracts were prepared using IP lysis buffer (Pierce) containing Protease Inhibitor Cocktail (Roche, Basel, Switzerland) and Phosphatase Inhibitor Cocktail (Sigma-Aldrich). The protein concentration was estimated using a Bradford Assay kit (Bio-Rad, Hercules, CA, USA). Proteins were resolved on 6, 10, or 12% polyacrylamide gels and transferred to PVDF membranes using a wet transfer apparatus from Bio-Rad. The membranes were blocked with 5% skim milk and probed with primary antibodies followed by the appropriate ECL-grade secondary HRP-conjugated antibody (GE Healthcare, Chicago, IL, USA). The blots were developed using the Supersignal Pico or Femto Reagent (Pierce Biotechnology, Waltham, MA, USA), as necessary. The details of the antibodies are provided in Supplementary Table 8.

### MTT assay

For MTT assays, 3 × 10^3^ ovarian cancer cells (COV-413B, ADR-RES, OVCAR-3, IGROV-1, or SK-OV-3) were plated in triplicate in a volume of 100 µL on 96-well plates. After 48 h, EPZ-5676, EPZ004777, and SGC0946 were added as indicated. Cell viability was evaluated after 3 days of treatment. To measure cell viability, 20 µL 5 mg/mL MTT solution dissolved in 1× PBS was added to each well of the 96-well plate and incubated for 1 h at 37 °C. The MTT solution was then gently removed, and 100 µL DMSO was added to each well. After the contents of each well were mixed well by pipetting, absorbance was measured at 590 and 630 nm using the Biotek Synergy MX Multi Format Microplate Reader (Biotek, Winooski, VT, USA). The average absorbance at 630 nm was subtracted from the average absorbance at 590 nm, and the relative growth rate was plotted with respect to vehicle control treated cells.

### Soft agar assay

For the soft agar assay, 5 × 10^3^ ovarian cancer cells (COV-413B, ADR-RES, OVCAR-3, IGROV-1, or SK-OV-3) were seeded into a 0.4% soft agar layer. After 24 h, the cells were treated with various concentrations of EPZ-5676, EPZ004777, and SGC0946. After 2–3 weeks, images of the colonies formed in the soft agar were taken using an inverted light microscope. The colonies were stained with 0.005% crystal-violet solution and counted. The average colony area of each sample was calculated using Image J software (NIH, Bethesda, MD, USA) and plotted. Each experiment was repeated at least twice.

### Mouse xenograft tumorigenesis and spontaneous metastasis experiments

IGROV-1 cells (10 × 10^6^) in Matrigel (Corning, Cat No. 356237, Corning, NY, USA) were injected subcutaneously into four female 5–6-week-old NSG mice per experimental group (Jackson Laboratory, Stock No. 005557, Bar Harbor, ME, USA). The tumor volume was then measured every week. Tumor size was calculated using the following formula: length × width^2^ × 0.5. Vehicle control (0.5% methyl cellulose in phosphate-buffered saline [PBS]) or EPZ-5676 or EPZ004777 (50 mg/kg body weight) were administered by intraperitoneal injection every day starting 1 week after the cells were injected (tumor volumes ∼80–100 mm^3^) until the end of the experimental period. After the start of EPZ-5676/EPZ004777 treatment, the tumor size was measured and plotted. At the end of the experiment, mice were sacrificed and subcutaneous tumors were harvested and imaged. All protocols for the mouse experiments were approved by the Institutional Animal Care and Use Committee of the University of Alabama at Birmingham.

### Apoptosis and necrosis measurements

IGROV-1 and SK-OV-3 cells were seeded at the density of 3000 cells in 75 µl media per well in white TC-treated clear-bottom 96-well plates (Costar Cat. No. #3610) and incubated for 24 h at 37 °C, 95% relative humidity, and 5% CO_2_. The cells were then treated with vehicle (DMSO) or 10 µM EPZ-5676 followed by immediate addition of Real Time-Glo Annexin V apoptosis and necrosis reagent (Cat. No. # JA1011, Promega Corp., Madison, WI, USA). Luminescence and fluorescence signals were monitored up to 24, 48, and 72 h using a Biotek Synergy MX Multi Format Microplate Reader.

### LDH cytotoxicity assay to measure NK cell-mediated eradication of ovarian cancer

The LDH cytotoxicity assay was performed using the LDH cytotoxicity assay kit from Thermo Fisher Scientific (cat. no. 88953), as previously described. NK92MI cells (1 × 10^6^ cells/ml; 100 μl) served as effector cells and were mixed at a 1:15 ratio in low-attachment round-bottom 96-well tissue culture plates (Costar Cat. No. #7007) with 10 × 10^3^ IGROV-1 cells that had been pretreated for 48 h with 10 µM vehicle or EPZ-5676. The plates were incubated at 37 °C in a CO_2_ incubator for 2 h. After incubation, the 96-well tissue culture plates were centrifuged at 1000 rpm for 3 min. The supernatants were then collected from each well and transferred into a fresh 96-well plate. Then, 50 μl LDH substrate mixture was added to each well. The plate was incubated for 10–20 min at room temperature in the dark, and absorbance at 490 and 680 nm was measured using a Biotek Synergy MX Multi Format Microplate Reader. The absorbance at 680 nm was subtracted from the absorbance at 490 nm to calculate the percent cytotoxicity using the following formula:

$$\frac{{{\mathrm{LDH}}\,{\mathrm{experimental}}-{\mathrm{LDH}}\,{\mathrm{effector}}\,{\mathrm{cells}}-{\mathrm{LDH}}\,{\mathrm{spontaneous}} {\,\times\, 100}}}{{{\mathrm{LDH}}\,{\mathrm{maximal}}-{\mathrm{LDH}}\,{\mathrm{spontaneous}}}}$$

### Metabolomic analysis

IGROV-1 cells treated with DOT1L inhibitor EPZ-5676 or control treated were analyzed for metabolic pathway alterations using the capillary electrophoresis time-of-flight mass spectrometry-based basic scan profiling method from Human Metabolome Technologies (Boston, MA, USA). Cells (1 × 10^6^) for each condition in duplicate were analyzed by this method, and samples were prepared as per the recommendations of Human Metabolome Technologies (Cambridge, MA, USA). For data analysis, peaks detected in capillary electrophoresis time-of-flight mass spectrometry analysis were extracted using automated integration software (MasterHands version 2.16.0.15 developed at Keio University, Tokyo, Japan) to obtain the mass/charge ratio (m/z), migration time, and peak area. Peak area was then converted to relative peak area using the following equation: relative peak area = metabolite peak area/internal standard peak area × number of cells. The peak detection limit was determined based on a signal-to-noise ratio = 3. Putative metabolites were then assigned from the Human Metabolomic Technologies standard library and the known–unknown peak library on the basis of m/z and migration time. All metabolite concentrations were calculated by normalizing the peak area of each metabolite with respect to the area of the internal standard and then using standard curves, which were obtained by single-point (100 μm) calibrations. The profile of peaks of putative metabolites was represented on metabolic pathway maps using Visualization and Analysis of Networks containing Experimental Data (VANTED) software (http://vanted.ipk-gatersleben.de/).

### Statistical Analysis

All experiments were conducted with at least three biological replicates. Results for individual experiments are expressed as the mean ± standard error of the mean. Measurements of tumor progression in mice and MTT assays were compared using the area under the curve method in the GraphPad Prism software, version 7.0, for Macintosh (GraphPad Software; https://www.graphpad.com). For the remaining experiments, *p* values were calculated using two-tailed unpaired Student’s *t*-tests in the GraphPad Prism software, version 7.0, for Macintosh.

## Supplementary information

Supplemental data

Supplementary Table 1

Supplementary Table 2

Supplementary Table 3

Supplementary Table 4

Supplementary Table 5

Supplementary Table 6

Supplementary Table 7

Supplementary Table 8
