# Supplementary material for: Disruptor of telomeric silencing 1-like promotes ovarian cancer tumor growth by stimulating pro-tumorigenic metabolic pathways and blocking apoptosis
Source: Oncogenesis. 2021 Jul 12;10(7):48. doi: 10.1038/s41389-021-00339-6 (PMC8275629; doi:10.1038/s41389-021-00339-6)
Supplement: Supplementary file 1 — Supplemental data [file 41389_2021_339_MOESM1_ESM.pdf]

SUPPLEMENTARY FIGURES and LEGENDS

Supplementary Fig. 1

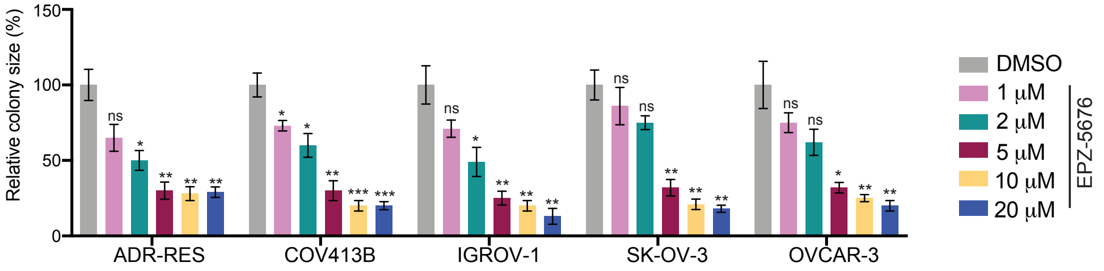

Supplementary Fig. 1. EPZ-5676 inhibits the growth of ovarian cancer cells. The indicated ovarian cancer cell lines were treated with various concentrations of EPZ-5676 and then analyzed for their ability to grow in an anchorage-independent manner in soft agar to determine the relative colony size. Representative images of soft agar assays are shown in Fig 3B. Data are shown as the mean ± SEM, *p<0.05, *p<0.01, ***p < 0.001, calculated using the Student's t-test.

Supplementary Fig. 2

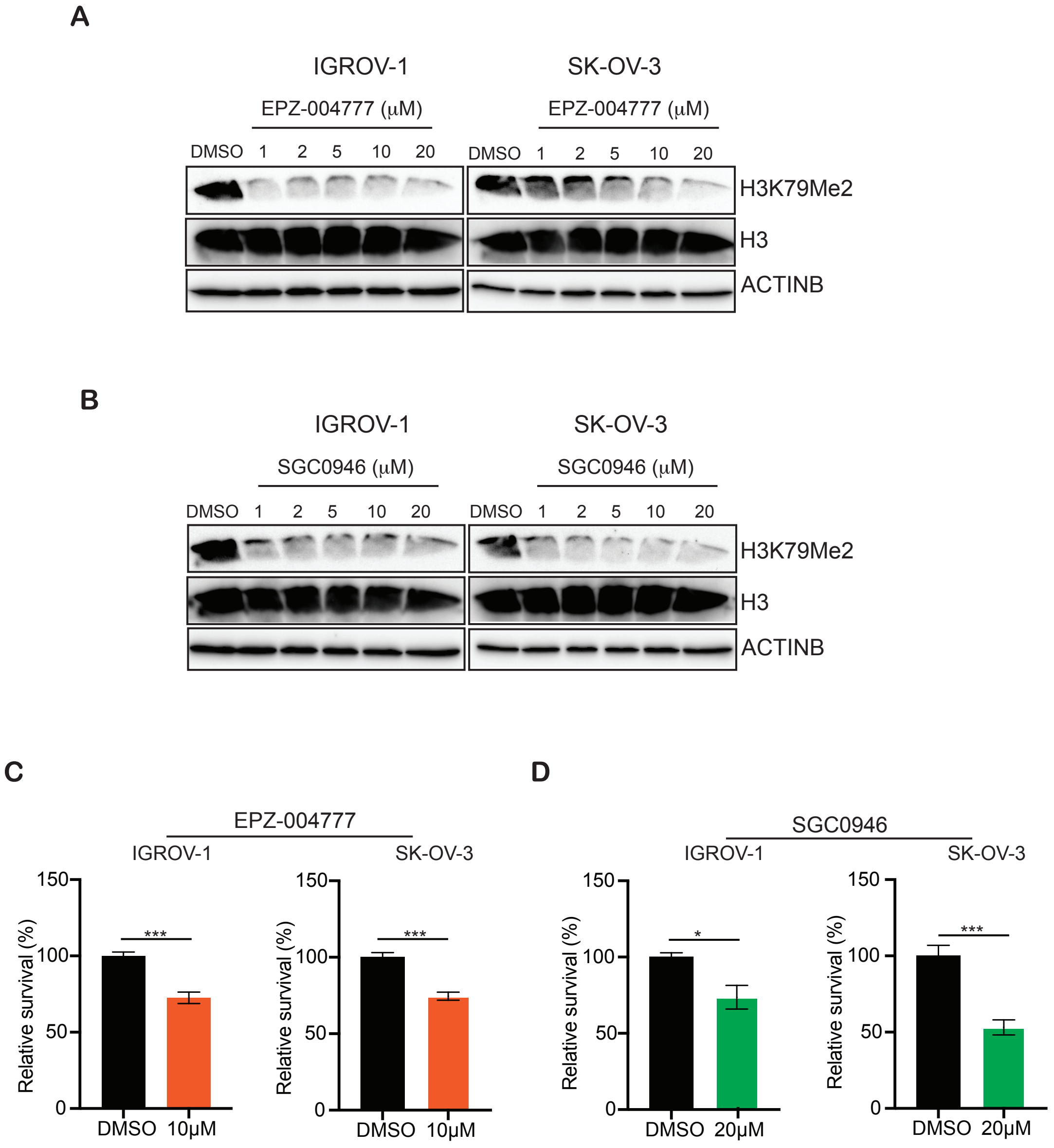

Supplementary Fig. 2. Pharmacological inhibition of DOT1L attenuates the growth of ovarian cancer cells. **A-B**. The indicated ovarian cancer cell lines were treated with various concentrations of DOT1L inhibitors EPZ004777 and SGC0946 for 48 h. H3K79dimethyl marks were then measured. Histone H3 and ACTINB proteins were measured as loading controls. **C-D**. The indicated ovarian cancer cell lines were treated with the DOT1L inhibitor EPZ-004777 (10 μM) and SGC0946 (20 μM) for 3 days and analyzed for cell survival in MTT assays. Relative cell

survival is plotted with respect to control cells. Data are shown as the mean ± SEM, $*p<0.05$, $***p < 0.001$, calculated using the Student's t-test.

Supplementary Fig. 3

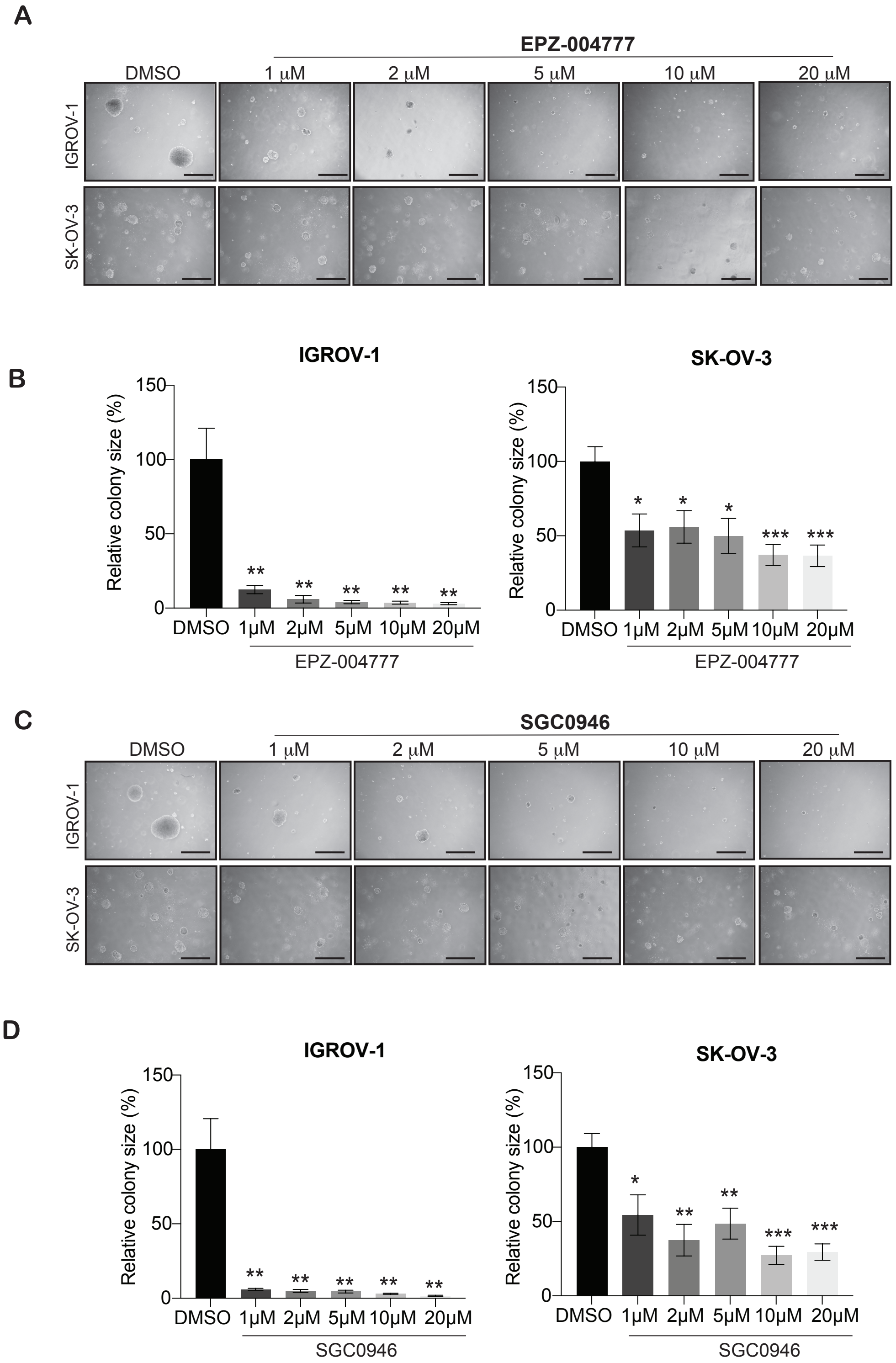

Supplementary Fig. 3. EPZ-004777 and SGC0946 inhibit the growth of ovarian cancer cells.

A. The indicated ovarian cancer cell lines were treated with various concentrations of EPZ-004777

and analyzed for their ability to grow in an anchorage-independent manner in soft agar. Representative images of soft agar assays are shown. Scale bar, 500 μm. **B**. Relative colony size for the soft agar images shown in panel A. **C.** The indicated ovarian cancer cell lines were treated with various concentrations of SGC0946 and analyzed for their ability to grow in an anchorage-independent manner in soft agar. Representative images of soft agar assays are shown. Scale bar, 500 μm**. D**. Relative colony size for the soft agar images shown in panel C. Data are shown as the mean ± SEM, *p<0.05, **p<0.01, ***p < 0.001, ns = not significant, calculated using the Student's t-test.

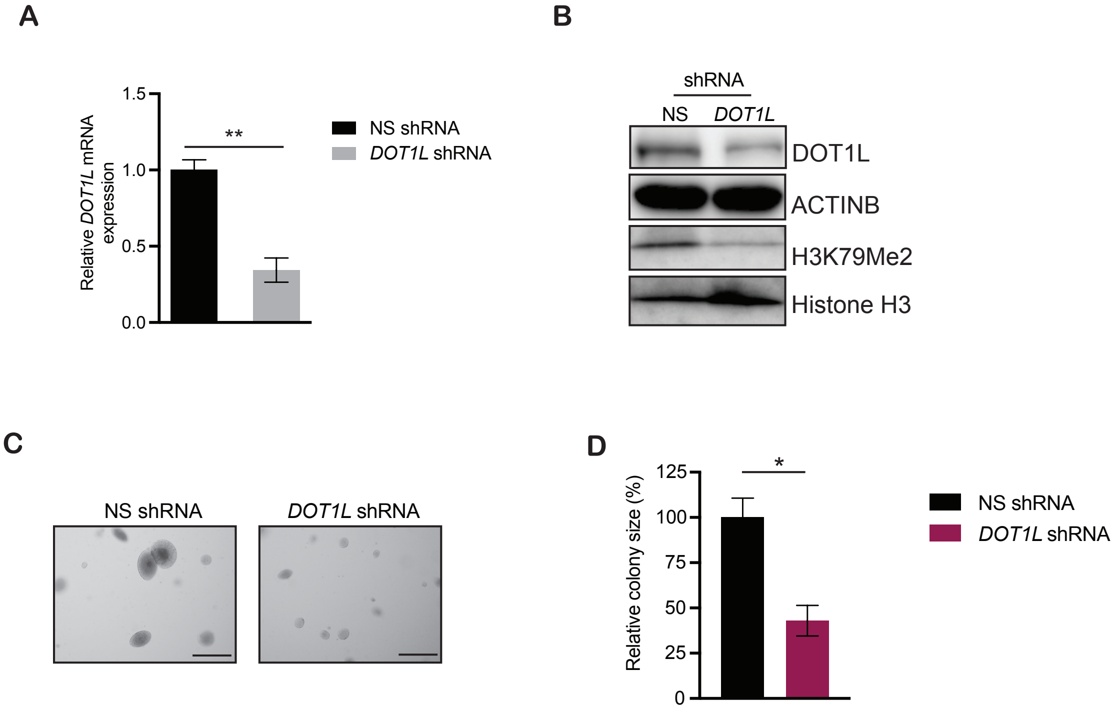

Supplementary Fig. 4. DOT1L knockdown inhibits the growth of ovarian cancer cells. **A.** IGROV-1 cell lines expressing either *DOT1L* or NS shRNA were analyzed for DOT1L expression by RT-qPCR. *DOT1L* mRNA expression in *DOT1L* shRNA-expressing cells relative to NS shRNA expressing cells is shown. ACTB was used for normalization. **B.** Immunoblotting of DOT1L and H3K79dimethyl marks in IGROV-1 cell lines expressing *DOT1L* or NS shRNA. Histone H3 and ACTINB proteins were measured as loading controls. **C.** The indicated IGROV-1 cell lines expressing either *DOT1L* or NS shRNA were analyzed in the soft agar assay. Representative images of soft agar colony formation are shown**.** Scale bar, 500 μm. **D.** Relative soft agar colony size for the data shown in panel C. Data are shown as the mean ± SEM, *p<0.05, **p < 0.01, ns = not significant, calculated using the Student's t-test.

Supplementary Fig. 5

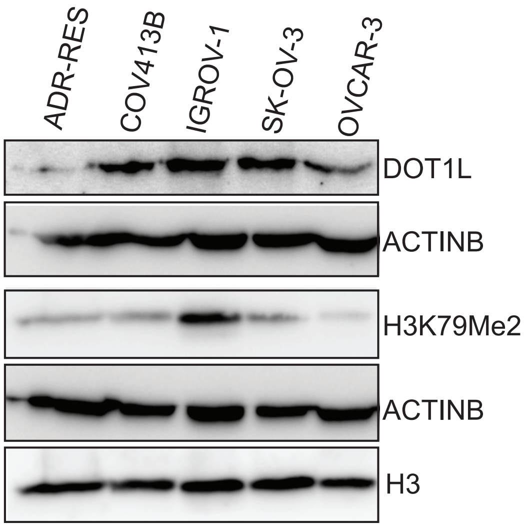

Supplementary Fig. 5. DOT1L expression in various ovarian cancer cells. Indicated ovarian cancer cell lines were analyzed for the following proteins and ACTINB and Histone H3 proteins were used as loading controls.

Supplementary Fig. 6

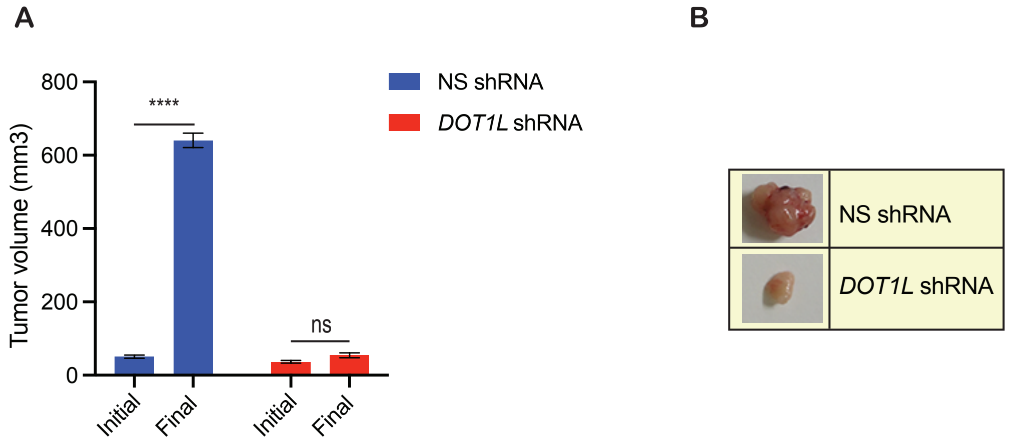

Supplementary Fig. 6. DOT1L knockdown inhibits the growth of ovarian cancer cells in vivo

A-B. IGROV-1 cell lines expressing either *DOT1L* or NS shRNA were injected subcutaneously into the flanks of NSG mice (n = 6) and analyzed for tumor growth. Tumor sizes were measured and the average tumor volume at the initial and final timepoints is plotted (**A**). Representative images of the tumors at the final time point are shown in (**B**). Data are shown as the mean ± SEM, ****$p<0.01$, ns = not significant, calculated using the Student's t-test.

Supplementary Fig. 7

A

Common Upregulated Genes
[IGR_tr_v_untr_U] and [SK_tr_v_untr_U]:

ADGRG1	HKDC1	PEA15	SPHK1
AKR1B10	HLA-B	PFDN2	SQSTM1
AKR1C2	HLA-F	PHGDH	SRXN1
AKR1C3	IL1A	PID1	STC2
AREG	IL32	PIM3	STK40
ATF3	INHBE	PLAT	SYT1
BHLHE40	IRF1	POLR2F	SYTL3
BMP2	IRF8	POLR3K	TIMM17B
CCND2	IRS2	PPP1R14C	TMEM54
CHAC1	JDP2	PPP1R15A	TMSB10
CLDN1	JUN	PSG9	TMSB4X
COX17	LAMC2	PTGS1	TNFAIP3
CPE	LARP6	RAB6B	TNFRSF21
DAPK2	LIF	RASGRP1	TRIB3
DDIT3	LOC103021296	RBP1	TSPAN12
DDIT4	MAFF	RND3	TUBB2A
DRAP1	MAP1B	RRP9	TWF2
DYNLT1	MARS	S1PR3	TXNRD1
ENPP5	MRPL52	SAA1	UBL5
FTL	MT2A	SERPINE1	UNC5B
FZD8	NKAIN1	SIPA1L2	VEGFA
GADD45A	NOV	SLC1A4	WNT6
GSTP1	NQO1	SLC3A2	
H3F3B	OSGIN1	SLC7A5	
HES1	PCK2	SLPI	

B

Common Downregulated Genes
[IGR_tr_v_untr_D] and [SK_tr_v_untr_D]

ADGRL2	FAM210B	LRRC41	SLC16A10
AMIGO2	FAM8A1	MAGT1	SMIM14
ARID5B	GPRC5A	MECOM	SPATS2L
BICC1	HMGA2	MEIS2	STARD13
C1R	HOXB2	MIER1	STON2
CALCOCO1	HOXB3	NLGN4X	TFAP2A
CCNYL2	HOXB4	NR2F2	UACA
CD68	HOXB5	NRP2	UCP2
CGNL1	HSDL2	PDGFC	VAV3
CHD3	IGF2BP2	PLOD1	ZFP36L2
CKB	IPMK	PLS3	ZMYM3
COL1A2	ITGB4	PTPRM	ZMYND8
DBNDD1	KCTD1	RAB26	ZNF217
DDHD2	KIRREL	RASSF3	ZNF532
DPP4	LAMB1	RBPJ	
DPYD	LBH	RFX7	
EHF	LGR4	SCNN1A	
EZH2	LOX	SDC3	

Supplementary Fig. 7. RNA sequencing identified common genes that are altered in IGROV-1 and SK-OV-3 cells upon DOT1L inhibition via EPZ-5676.

A. Ninety-seven genes were upregulated in IGROV-1 (IGR_tr_v_untr_U) and SK-OV-3 (SK_tr_v_untr_U) cells after treatment with 10 μM EPZ-5676 for 48 h. **B.** Sixty-eight genes were downregulated genes in IGROV-1 (IGR_tr_v_untr_U) and SK-OV-3 (SK_tr_v_untr_U) cells after treatment with 10 μM EPZ-5676 for 48 h.

Gene Ontology Biological Process : Upregulated Pathways

geneSet	Description	size	Overlap	Expect	ER	pValue	FDR	Gene Symbols
GO:1902531	regulation of intracellular signal transduction	1824	29	9.96	2.91	6.02E-08	0.00055	ADGRG1; AKR1C2; AKR1C3; ATF3; BMP2; DAPK2; DDIT3; DDIT4; DYNLT1; FZD8; GADD45A; GSTP1; HES1; INHBE; IRS2; JUN; LIF; PEA15; RASGRP1; SAA1; SIPA1L2; SPHK1; SQSTM1; STK40; TMSB4X; TNFAIP3; TRIB3; UNC5B; VEGFA
GO:0006915	apoptotic process	1912	29	10.44	2.78	1.68E-07	0.00076	AKR1C3; ATF3; BMP2; CCND2; CHAC1; DAPK2; DDIT3; DDIT4; GADD45A; GSTP1; IL1A; INHBE; IRF1; IRS2; JUN; NQO1; OSGIN1; PEA15; PIM3; PPP1R15A; SERPINE1; SPHK1; SQSTM1; STK40; TNFAIP3; TNFRSF21; TRIB3; UNC5B; VEGFA
GO:0019220	regulation of phosphate metabolic process	1690	26	9.23	2.82	6.98E-07	0.00162	AREG; ATF3; BMP2; CCND2; DDIT4; FZD8; GADD45A; GSTP1; HES1; INHBE; IRS2; JUN; LIF; PEA15; PID1; PPP1R14C; PPP1R15A; RASGRP1; SAA1; SPHK1; SQSTM1; STK40; TMSB4X; TNFAIP3; TRIB3; VEGFA
GO:0051174	regulation of phosphorus metabolic process	1692	26	9.24	2.81	7.14E-07	0.00162	AREG; ATF3; BMP2; CCND2; DDIT4; FZD8; GADD45A; GSTP1; HES1; INHBE; IRS2; JUN; LIF; PEA15; PID1; PPP1R14C; PPP1R15A; RASGRP1; SAA1; SPHK1; SQSTM1; STK40; TMSB4X; TNFAIP3; TRIB3; VEGFA
GO:0042325	regulation of phosphorylation	1500	24	8.19	2.93	1.06E-06	0.00193	AREG; ATF3; BMP2; CCND2; DDIT4; FZD8; GADD45A; GSTP1; HES1; INHBE; IRS2; JUN; LIF; PEA15; PID1; PPP1R14C; RASGRP1; SAA1; SPHK1; SQSTM1; STK40; TNFAIP3; TRIB3; VEGFA
GO:0008284	positive regulation of cell proliferation	898	18	4.90	3.67	1.40E-06	0.00208	ADGRG1; AKR1C2; AKR1C3; AREG; ATF3; BMP2; CCND2; COX17; HES1; IRS2; JUN; LAMC2; LIF; PID1; S1PR3; SPHK1; TNFAIP3; VEGFA
GO:0010941	regulation of cell death	1649	25	9.00	2.78	1.60E-06	0.00208	AKR1C3; ATF3; BMP2; CCND2; DAPK2; DDIT3; DDIT4; DYNLT1; GADD45A; GSTP1; IL1A; INHBE; IRS2; JUN; NQO1; OSGIN1; PEA15; PIM3; SERPINE1; SPHK1; SQSTM1; STK40; TNFAIP3; UNC5B; VEGFA
GO:1902644	tertiary alcohol metabolic process	17	4	0.09	43.09	1.87E-06	0.00213	AKR1B10; AKR1C2; AKR1C3; BMP2
GO:0097190	apoptotic signaling pathway	582	14	3.18	4.41	3.08E-06	0.00311	ATF3; CHAC1; DAPK2; DDIT3; DDIT4; GSTP1; IL1A; JUN; PEA15; PPP1R15A; SERPINE1; TNFAIP3; TRIB3; UNC5B
GO:0001932	regulation of protein phosphorylation	1388	22	7.58	2.90	3.93E-06	0.00352	AREG; ATF3; BMP2; CCND2; DDIT4; FZD8; GADD45A; GSTP1; HES1; INHBE; JUN; LIF; PEA15; PID1; RASGRP1; SAA1; SPHK1; SQSTM1; STK40; TNFAIP3; TRIB3; VEGFA

Supplementary Fig. 8. Pathway analysis of common upregulated genes in IGROV-1 and SK-OV-3 cells upon DOT1L inhibition via EPZ-5676.

Common upregulated genes (68) in IGROV-1 and SK-OV-3 cells upon DOT1L inhibition via EPZ-5676 were analyzed using gene ontology biological processes. The description of each pathway and the genes participating in each pathway are shown along with the p-value and false discovery rate.

Supplementary Fig. 9

Gene Ontology Biological Process : Downregulated Pathways

geneSet	description	size	overlap	expect	ER	pValue	FDR	Gene Symbols
GO:0021602	cranial nerve morphogenesis	28	4	0.10	39.03	3.11E-06	0.0214	HOXB2; HOXB3; NRP2; TFAP2A
GO:0009887	animal organ morphogenesis	974	14	3.56	3.93	8.42E-06	0.0214	ARID5B; COL1A2; HOXB2; HOXB3; HOXB4; HOXB5; ITGB4; LAMB1; LGR4; NRP2; PDGFC; PTPRM; RBPJ; TFAP2A
GO:0010558	negative regulation of macromolecule biosynthetic process	1434	17	5.25	3.24	9.77E-06	0.0214	ARID5B; EZH2; HMGA2; HOXB3; HOXB4; IGF2BP2; KCTD1; LGR4; MECOM; MEIS2; MIER1; NR2F2; RBPJ; TFAP2A; ZFP36L2; ZMYND8; ZNF217
GO:0001501	skeletal system development	506	10	1.85	5.40	1.37E-05	0.0214	ARID5B; COL1A2; HMGA2; HOXB2; HOXB3; HOXB4; HOXB5; PDGFC; PLS3; TFAP2A
GO:0031327	negative regulation of cellular biosynthetic process	1492	17	5.46	3.11	1.65E-05	0.0214	ARID5B; EZH2; HMGA2; HOXB3; HOXB4; IGF2BP2; KCTD1; LGR4; MECOM; MEIS2; MIER1; NR2F2; RBPJ; TFAP2A; ZFP36L2; ZMYND8; ZNF217
GO:0009890	negative regulation of biosynthetic process	1516	17	5.55	3.06	2.03E-05	0.0214	ARID5B; EZH2; HMGA2; HOXB3; HOXB4; IGF2BP2; KCTD1; LGR4; MECOM; MEIS2; MIER1; NR2F2; RBPJ; TFAP2A; ZFP36L2; ZMYND8; ZNF217
GO:2000113	negative regulation of cellular macromolecule biosynthetic process	1359	16	4.97	3.22	2.09E-05	0.0214	ARID5B; EZH2; HMGA2; HOXB3; HOXB4; IGF2BP2; KCTD1; LGR4; MECOM; MEIS2; MIER1; NR2F2; RBPJ; TFAP2A; ZFP36L2; ZNF217
GO:0048704	embryonic skeletal system morphogenesis	93	5	0.34	14.69	2.26E-05	0.0214	HOXB2; HOXB3; HOXB4; HOXB5; TFAP2A
GO:1903507	negative regulation of nucleic acid-templated transcription	1215	15	4.45	3.37	2.32E-05	0.0214	ARID5B; EZH2; HMGA2; HOXB3; HOXB4; KCTD1; LGR4; MECOM; MEIS2; MIER1; NR2F2; RBPJ; TFAP2A; ZMYND8; ZNF217
GO:1902679	negative regulation of RNA biosynthetic process	1217	15	4.45	3.37	2.37E-05	0.0214	ARID5B; EZH2; HMGA2; HOXB3; HOXB4; KCTD1; LGR4; MECOM; MEIS2; MIER1; NR2F2; RBPJ; TFAP2A; ZMYND8; ZNF217

Supplementary Fig. 9. Pathway analysis of common downregulated genes in IGROV-1 and SK-OV-3 cells upon DOT1L inhibition via EPZ-5676.

Common downregulated genes (97) in IGROV-1 and SK-OV-3 cells upon DOT1L inhibition via EPZ-5676 were analyzed using gene ontology biological processes. The description of each pathway and the genes participating in each pathway are shown with the p-value and false discovery rate.

Supplementary Fig. 10

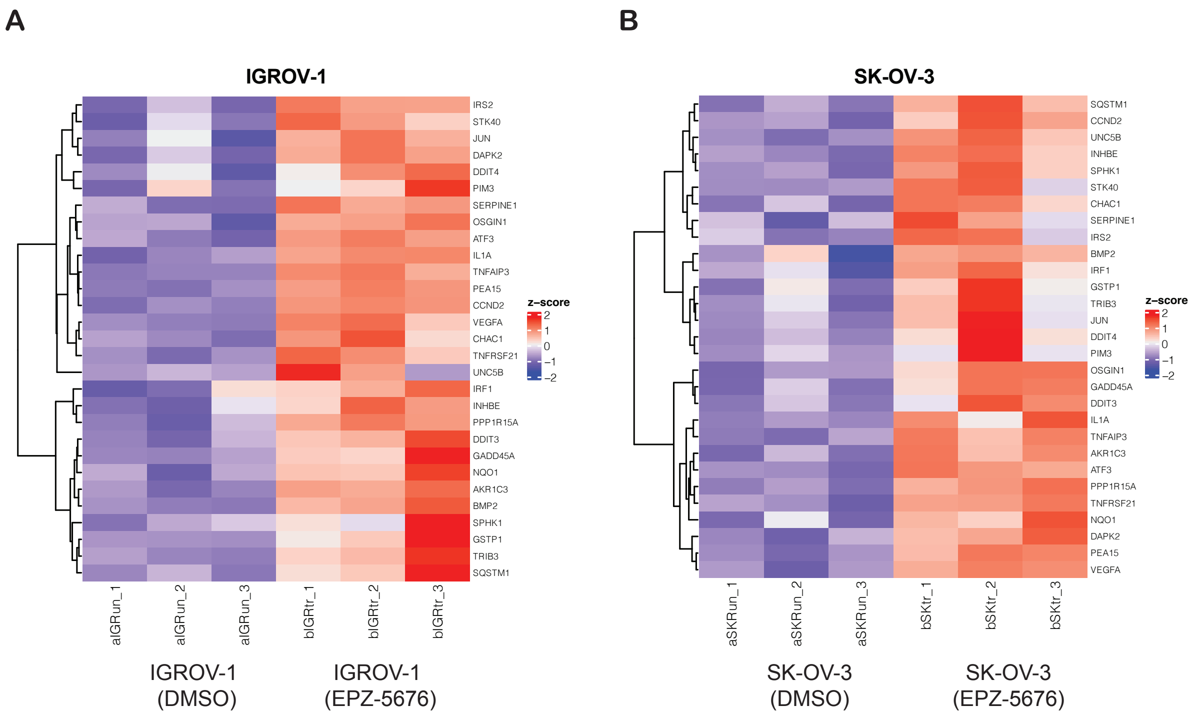

Supplementary Fig. 10. A-B. A heatmap showing the expression of candidate genes involved in apoptotic and cell-death pathways that were differentially expressed in IGROV-1 and SK-OV-3 cells after treatment with 10 μM EPZ-5676 for 48 h in comparison with control cells.

Supplementary Fig. 11

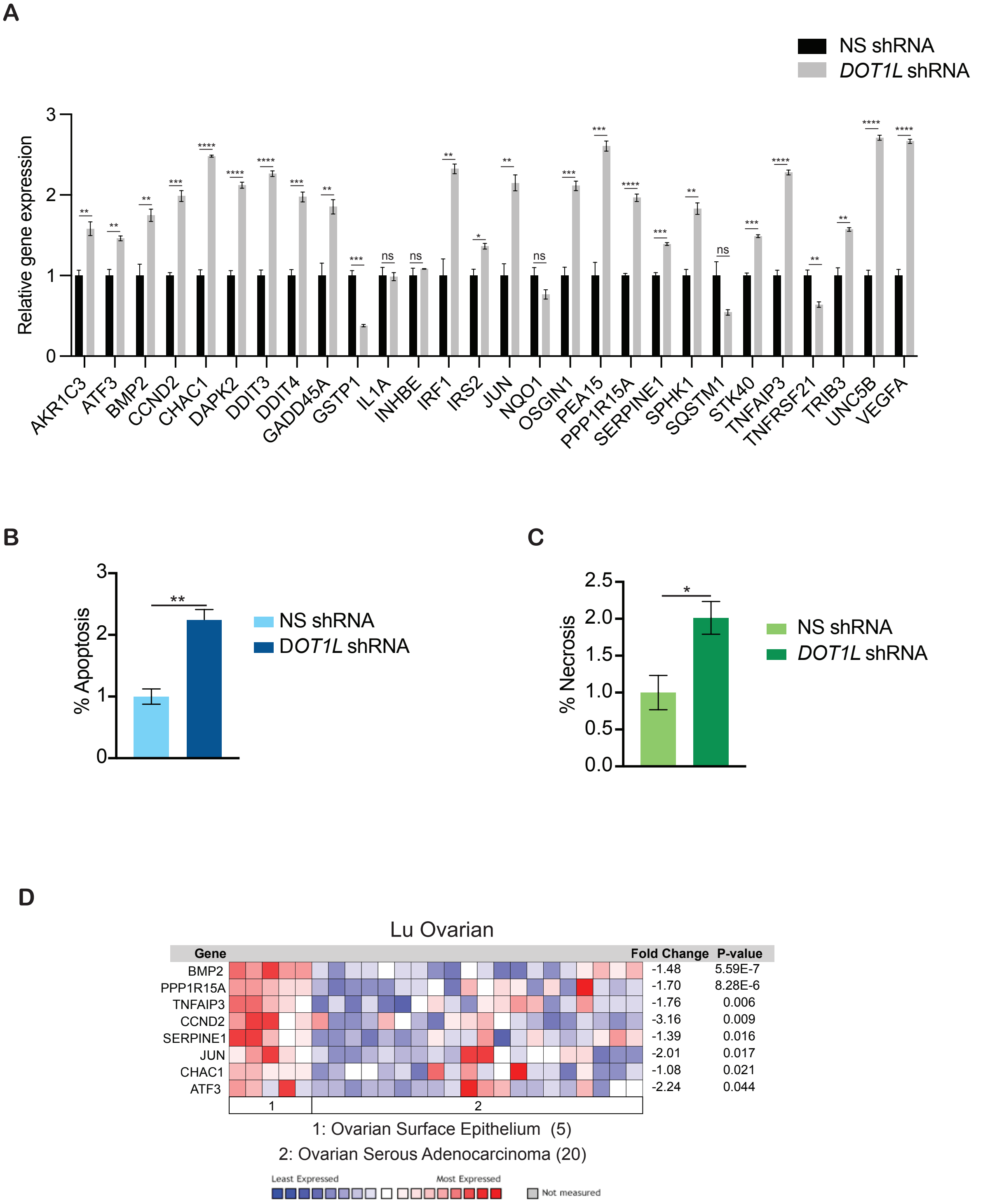

Supplementary Fig. 11. DOT1L knockdown leads to upregulation of multiple genes involved in apoptotic and cell-death pathways.

A. Expression of candidate genes involved in apoptotic and cell-death pathways were measured in IGROV-1 cell lines expressing *DOT1L* or NS shRNA. mRNA expression in *DOT1L* shRNA-expressing cells relative to NS shRNA-expressing cells is shown. ACTB was used for normalization. **B.** Bar diagram showing apoptosis in IGROV-1 cell lines expressing *DOT1L* or NS shRNA. **C.** Bar diagram showing necrosis in IGROV-1 cell lines expressing *DOT1L* or NS shRNA. **D.** The Lu ovarian cancer patient datasets were analyzed for apoptotic gene expression. The fold expression of candidate genes with p-values < 0.05 are shown. Data are shown as the mean ± SEM, *p<0.05, **p<0.01, ***p < 0.001, ****p < 0.0001, ns = not significant, calculated using the Student's t-test.

Supplementary Fig. 12

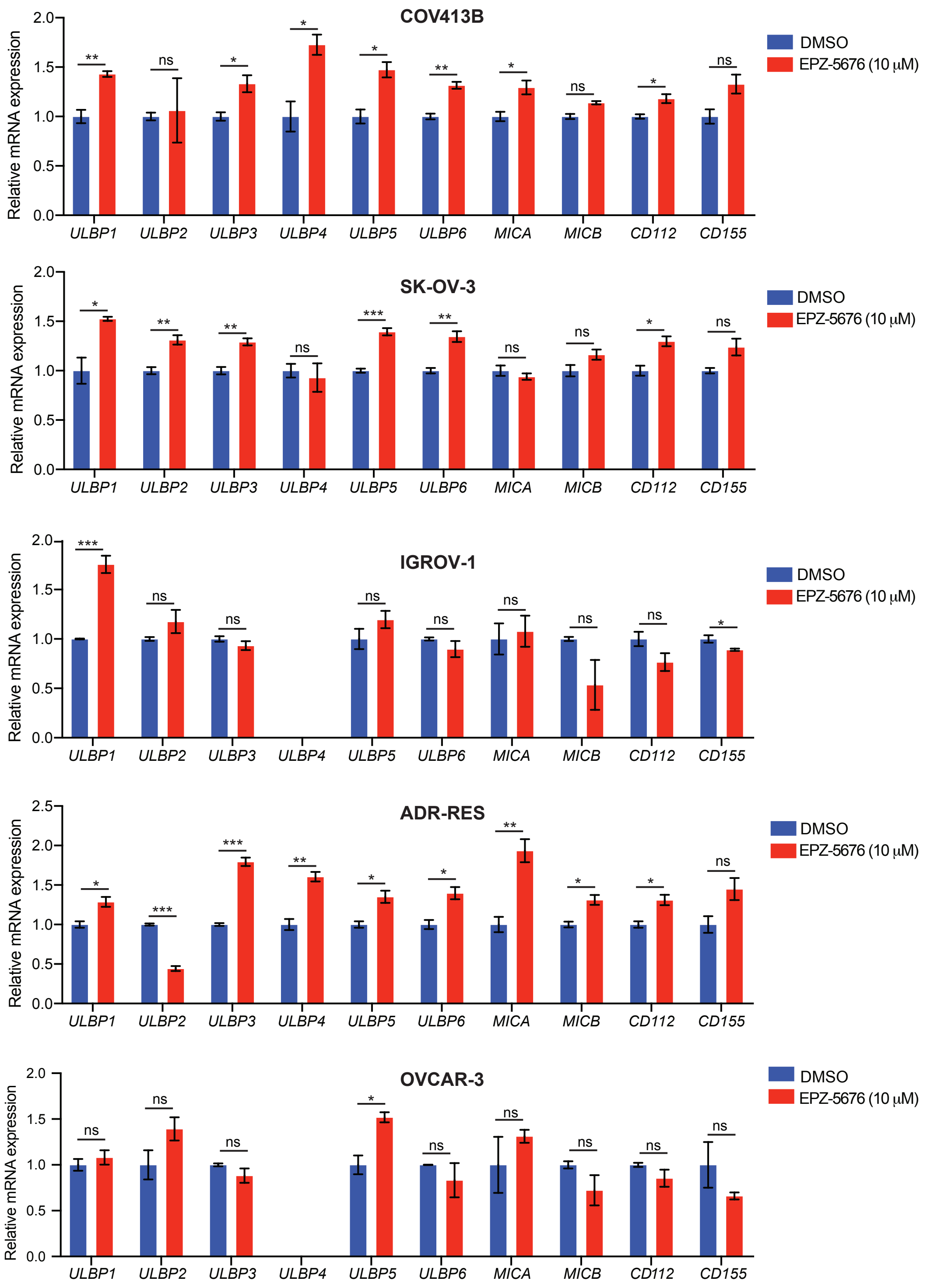

Supplementary Fig. 12. Measurement of NKG2D ligand expression in ovarian cancer cells after DOT1L inhibition via EPZ-5676.

The indicated ovarian cancer cells were treated with the DOT1L inhibitor EPZ-5676 (10 μM) for 48 h and analyzed for expression of various NKG2D ligands. Data are plotted for the treated cells with respect to control cells. Data are shown as the mean ± SEM, $^*p<0.05$, $^{**}p<0.01$, $^{***}p < 0.001$, ns = not significant, calculated using the Student's t-test.

Supplementary Fig. 13

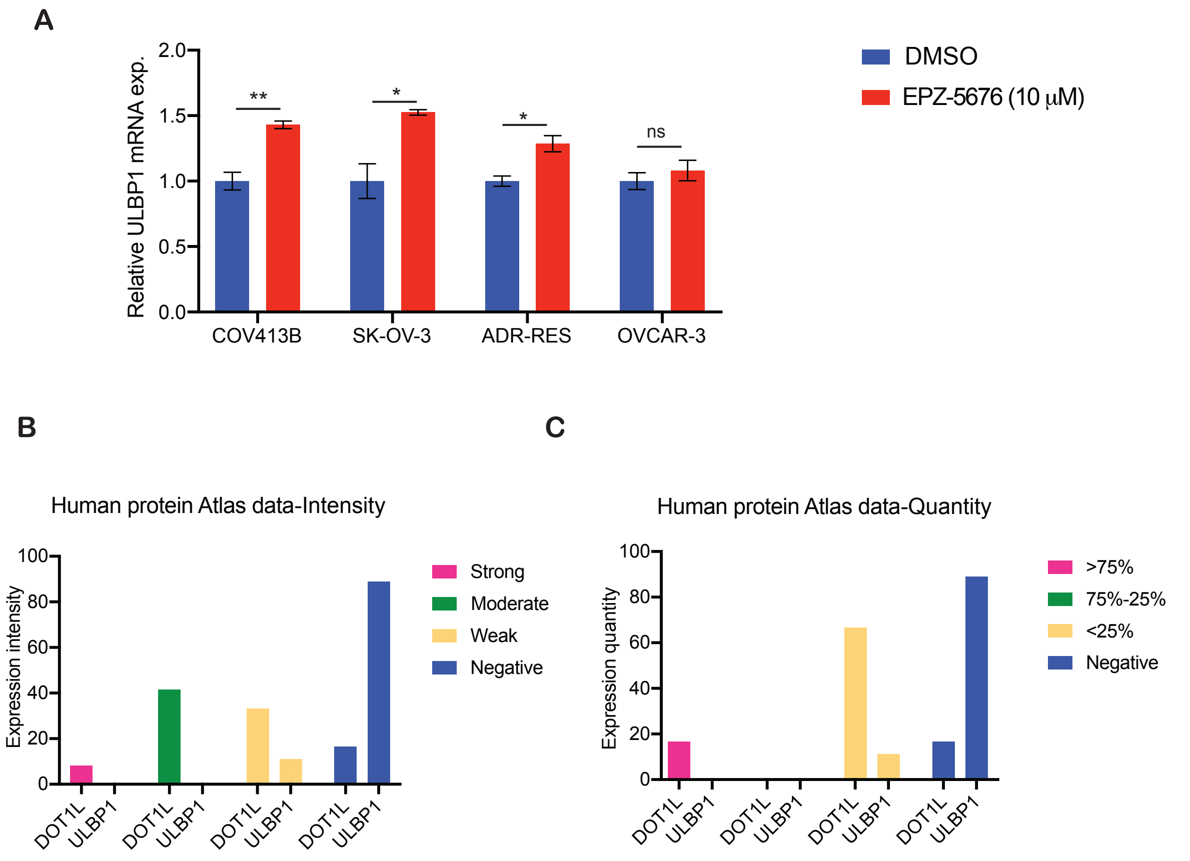

Supplementary Fig. 13. Measurement of ULBP1 levels in ovarian cancer cells after DOT1L inhibition via EPZ-5676.

A. The indicated ovarian cancer cell lines were treated with the DOT1L inhibitor EPZ-5676 (10 μM) for 48 h and analyzed for ULBP1 mRNA levels. Actin was used as an internal control. **B-C.** DOT1L and ULBP1 expression was analyzed using the human protein atlas data set. The relative intensity (**B**) and quantity (**C**) of expression are plotted and compared. Data are shown as the mean ± SEM, $*p<0.05$, $**p<0.01$, ns = not significant, calculated using the Student's t-test.

SUPPLEMENTARY TABLES

Supplementary Table 1: Significant differentially expressed genes in EPZ-5676 treated IGROV-1 cells. RNA sequencing data showing significant differentially expressed gene in IGROV-1 cell upon treatment with DOT1L inhibitor EPZ-5676 in comparison to control treated cells.

Supplementary Table 2: Significant differentially expressed genes in EPZ-5676 treated SK-OV-3 cells. RNA sequencing data showing significant differentially expressed gene in SK-OV-3 cell upon treatment with DOT1L inhibitor EPZ-5676 in comparison to control treated cells.

Supplementary Table 3: Common significant differentially expressed genes in EPZ-5676 treated IGROV-1 cells. RNA sequencing data showing common significant differentially expressed gene in IGROV-1 cell upon treatment with DOT1L inhibitor EPZ-5676 in comparison to control treated cells.

Supplementary Table 4: Common significant differentially expressed genes in EPZ-5676 treated SK-OV-3 cells. RNA sequencing data showing common significant differentially expressed gene in SK-OV-3 cell upon treatment with DOT1L inhibitor EPZ-5676 in comparison to control treated cells.

Supplementary Table 5: Significantly upregulated pathways in EPZ-5676 treated ovarian cancer cells. Pathway analysis performed using RNA sequencing data showing common

significant upregulated gene in IGROV-1 and SK-OV-3 cell upon treatment with DOT1L inhibitor EPZ-5676 in comparison to control treated cells with full name of genes identified in RNA sequencing data.

Supplementary Table 6: Significantly downregulated pathways in EPZ-5676 treated ovarian cancer cells. Pathway analysis performed using RNA sequencing data showing common significant downregulated gene in IGROV-1 and SK-OV-3 cell upon treatment with DOT1L inhibitor EPZ-5676 in comparison to control treated cells with full name of genes identified in RNA sequencing data.

Supplementary Table 7: Metabolomics analysis in EPZ-5676 treated ovarian cancer IGROV-1 cell. Metabolomics analysis showing metabolites altered in IGROV-1 cell upon treatment with DOT1L inhibitor EPZ-5676 in comparison to control treated cells.

Supplementary Table 8: List of Reagents, data and software used in this study with source and identifier.
