# Supplementary material for: Disruptor of telomeric silencing 1-like promotes ovarian cancer tumor growth by stimulating pro-tumorigenic metabolic pathways and blocking apoptosis
Source: Oncogenesis. 2021 Jul 12;10(7):48. doi: 10.1038/s41389-021-00339-6 (PMC8275629; doi:10.1038/s41389-021-00339-6)
Supplement: Supplementary file 9 — Supplementary Table 8 [file 41389_2021_339_MOESM9_ESM.docx]

**Supplementary Table 9.** List of Reagents, data and software used in this study with source and identifier.

| **REAGENT or RESOURCE** | **SOURCE** | **IDENTIFIER** |
| --- | --- | --- |
| Antibodies | | |
| DOT1L | Cell Signaling | Cat# 90878S |
| H3K79Me2 | Cell Signaling | Cat# 5427S |
| ACTINB | Cell Signaling | Cat#4970S |
| ULBP1 | Abcam | AB176566 |
| Chemicals, Peptides, and Recombinant Proteins |  |  |
| DMEM | GIBCO | Cat# 11965-092 |
| RPMI | GIBCO | Cat# 11875-093 |
| Fetal Bovine Serum | GIBCO | Cat# 10437-028 |
| Trypsin-EDTA | GIBCO | Cat# 25200-056 |
| Penicillin-Streptomycin | GIBCO | Cat# 15140-122 |
| Agarose, Low gelling | Sigma-Aldrich | Cat# A9414-100g |
| Deposited Data |  |  |
| RNA-Seq performed with IGROV-1 cells treated with EPZ-5676 | This paper | GEO:  GSE158019 |
| RNA-Seq performed with SK-OV3 cells treated with EPZ-5676 | This paper | GEO:  GSE158019 |
|  |  |  |
| Experimental Models: Cell Lines |  |  |
| OVCAR-3 | ATCC | ATCC^®^ HTB-161^™^ |
| ADR-RES | EZBiosystem | EZT-OVC8-1 |
| COV413B | Millipore Sigma | 07071906 |
| IGROV-1 | Millipore Sigma | SCC203 |
| SK-OV3 | Millipore Sigma | 91091004 |
| Experimental Models: Organisms/Strains |  |  |
| Mouse: female NSG | Jackson Laboratory | Stock No. 005557 |
| Oligonucleotides | | |
| **Gene name** | **Forward primer sequence** | **Reverse primer sequence** |
| *CD112* | acggtcacctgcaaagtgga | acggccgaggtaccagttgt |
| *CD155* | tgtcccgtaacgccatcatc | ccaaaggacctcacgggaac |
| *MICA* | cctgcaatcccagcactttg | attcaccaccaagcccgtct |
| *MICB* | cacgttcgccctttgttcag | ggaggcagaggttgcagtga |
| *ULBP1* | ccaccaggactggcaaactg | attgggaggccaaggtggta |
| *ULBP2* | caggcacaacccaactcagg | gccagacagaagggcgagtt |
| *ULBP3* | cctcgcgattcttccgtacc | Gccccacctctctcagcat |
| *ULBP4* | tcgccaccaatggagagaaa | attgcctcccagtgccctaa |
| *ULBP5* | gcttctgctcctgctgtcca | gggactgacgggtgtgactg |
| *ULBP6* | gccatgtcctcaggcacaac | tcagatgccagggaggatga |
| *ACTINB* | gcatggagtcctgtggcatc | Ttctgcatcctgtcggcaat |
| *AKR1C3* | actggccatccgaagcaaga | cttctccatggcctcccagg |
| *ATF3* | tgcccgcctttcatctggat | tggccgttgcacaacttcac |
| *BMP2* | tgtggagggttgtgggtgtc | aagtgtcaactggggtgggg |
| *CCND2* | aagtggaacctggcagctgt | ggcatcacaagtgagcgagc |
| *CHAC1* | gcgtctggcagacttcatgc | tgcactggagtgggtgtctg |
| *DAPK2* | agagtctggggtgggagtgt | ttggccaggctggttctgaa |
| *DDIT3* | cttgttccagccactcccca | ctggctcctcctcagtcagc |
| *DDIT4* | gctgcgtttaagccttcccc | gacccagatggcactcctcc |
| *GADD45A* | ggacgaggacgacgacagag | gattcgtcaccagcacgcag |
| *GSTP1* | ggcaggagggctcactcaaa | ccacgccgtcattcaccatg |
| *IL1A* | ctaagccttcctgccgcaac | tggaggatgcctggtcacac |
| *INHBE* | gctagcaagaggacctgggg | gccaggtggttgttgggttg |
| *IRF1* | agtggaagttgtgccggaca | cacgtttgttggctgccact |
| *IRS2* | cctcgtcctcctcgctcttg | ccggcttaggagacttgggg |
| *JUN* | ttcctgtgccccaagaacgt | cgcctgggttgaagttgctg |
| *NQO1* | caccgagagcctagttccgg | cgactccaccacctcccatc |
| *OSGIN1* | tcccggtgatccatgccttc | gaggctgcggtaaccctcat |
| *PEA15* | tttgagatctcccgccgtcc | ctcttcctcagagggctgcc |
| *PPP1R15A* | ccagtgatgggcctcctcag | ggggattgccagaggagtcc |
| *SERPINE1* | gagagagacaggcagctcgg | ttgcccaggctggtcttgaa |
| *SPHK1* | ggggagatgcgcttcactct | cagatgcatgacgccagctg |
| *SQSTM1* | ggatgaggacggggacttgg | cgcagatcacattggggtgc |
| *STK40* | tctacgacagcatcccgcag | gcaatgatggcactgaggg |
| *TNFRSF21* | tgtggaaaaggcagggctga | tccactggcttcccacttgg |
| *TRIB3* | cgaagccagggaagaggagg | agcacactccacacaggctt |
| *UNC5B* | ctacgtgcctctccctctgc | tgcaccaaagctcctggtca |
| *VEGFA* | ggaagagtagctcgccgagg | cctcctccttctgccatggg |
| *DOT1L* | tgaagagctcccctgtgccc | actgctgggctcgtcctcag |
|  |  |  |
| *shRNAs* |  |  |
| *DOT1L* | TRCN0000020209 | RHS3979-9587617 |
|  |  |  |
| Commercial assays and kits |  |  |
| RealTime-Glo™ Annexin V Apoptosis and Necrosis Assay | Promega | Cat# JA1011 |
|  |  |  |
| Inhibitors |  |  |
| EPZ-5676 | MedChemExpress | Cat# HY-15593 |
| EPZ-004777 | MedChemExpress | Cat# HY-15227 |
| SGC0946 | MedChemExpress | Cat# HY-15650 |
|  |  |  |
| Software and Algorithms | | |
| Prism 8.0 | GraphPad | www.graphpad.com/scientific software/prism |
| ImageJ | https://imagej.nih.gov/ij | N/A |
|  |  |  |
|  |  |  |
| Other |  |  |
| *DOT1L* mRNA expression in normal ovarian surface epithelium cells and ovarian cancer samples were analyzed and represented as box plot. | Oncomine Research Premium Edition | https://www.oncomine.org/ |
| *DOT1L* mRNA expression in ovarian cancer samples and PFS and OS survival was plotted using Km plotter | survival biomarker analysis tool | https://kmplot.com/analysis/ |
| Reactome pathway analysis from RNA sequencing data | Reactome Pathway Database | https://reactome.org/ |
